# Elucidating the Aromatic Properties of Covalent Organic Frameworks Surface for Enhanced Polar Solvent Adsorption

**DOI:** 10.3390/polym13111861

**Published:** 2021-06-03

**Authors:** Mostafa Yousefzadeh Borzehandani, Emilia Abdulmalek, Mohd Basyaruddin Abdul Rahman, Muhammad Alif Mohammad Latif

**Affiliations:** 1 Integrated Chemical BioPhysics Research, Faculty of Science, Universiti Putra Malaysia, UPM, Serdang 43400, Selangor, Malaysia; mostafa.yousefzadeh@gmail.com (M.Y.B.); emilia@upm.edu.my (E.A.); basya@upm.edu.my (M.B.A.R.); 2 Department of Chemistry, Faculty of Science, Universiti Putra Malaysia, UPM, Serdang 43400, Selangor, Malaysia; 3 Centre of Foundation Studies for Agricultural Science, Universiti Putra Malaysia, UPM, Serdang 43400, Selangor, Malaysia

**Keywords:** covalent organic frameworks, COF-1, CTF-1, aromaticity, stability, water, ethanol

## Abstract

Covalent organic frameworks (COFs) have a distinguished surface as they are mostly made by boron, carbon, nitrogen and oxygen. Many applications of COFs rely on polarity, size, charge, stability and hydrophobicity/hydrophilicity of their surface. In this study, two frequently used COFs sheets, COF-1 and covalent triazine-based frameworks (CTF-1), are studied. In addition, a theoretical porous graphene (TPG) was included for comparison purposes. The three solid sheets were investigated for aromaticity and stability using quantum mechanics calculations and their ability for water and ethanol adsorption using molecular dynamics simulations. COF-1 demonstrated the poorest aromatic character due to the highest energy delocalization interaction between B–O bonding orbital of sigma type and unfilled valence-shell nonbonding of boron. CTF-1 was identified as the least kinetically stable and the most chemically reactive. Both COF-1 and CTF-1 showed good surface properties for selective adsorption of water via hydrogen bonding and electrostatic interactions. Among the three sheets, TPG’s surface was mostly affected by aromatic currents and localized π electrons on the phenyl rings which in turn made it the best platform for selective adsorption of ethanol via van der Waals interactions. These results can serve as guidelines for future studies on solvent adsorption for COFs materials.

## 1. Introduction

Covalent organic frameworks (COFs) are well-known as two-dimensional (2D) and three-dimensional (3D) light porous crystalline [1,2,3]. COFs are constructed by organic building blocks that joined together through strong covalent bonds. Compared to other materials such as porous graphene, COFs have more polar surfaces provided by heteroatoms in the framework which offer a broad range of applications. For example, COF-105 and COF-108 have shown significant performance at 77 K towards hydrogen gas storage by 10.0 wt% at 80 bar and 10.0 wt% at 100 bar, respectively [4]. Additionally, COF-1 recorded 195 *v*/*v* (total volume per unit volume) at 30 bar and 298 K for methane storage which was higher than the U.S. Department of Energy target (180 *v*/*v* at 298 K and 35 bar) [5]. COFs have been studied as potential candidate for membrane separation in terms of its pore size, charge, stability, and hydrophobicity of the surface [6]. Consideration of these criteria is important in order to design relevant applications such as aqueous and solvent-based separation and adsorption.

Pore size is one of the immediate criteria to look upon when dealing with porous materials. There are two strategies for controlling the pore size in COFs. A desired pore size can be obtained by selecting a reasonable length of organic linkers [7]. Alternatively, different sizes of functional groups on linkers can be incorporated post-synthetically which can alter the pore size (post-synthetic modification) [8]. Free-standing covalent-organic-framework membranes, FS-COM-1 and FS-COM-2, have been synthesized and yielded a pore size of 0.6 and 1.1 nm, respectively [9]. These two COFs membranes underwent numerous separations and purifications tests. FS-COM-1 have been shown to facilely filter hydrogen ions (H^+^) while blocking larger ions or organic solvents due to its small pore size. A series of COFs were synthesized with three different lengths of organic linkers in the order of p-phenylenediamine (PDA) < benzidine (BD) < 4,4″-Diamino-p-terphenyl (DT) [10]. COF1, COF2 and COF3 have a pore size of 1.81, 2.57 and 3.34 nm, respectively. The results for adsorption study of triphenyl phosphate (TPhP) from aqueous solution showed that COF2 has the highest uptake efficiency due to proper pore size for TPhP (molecular size of 1.1 nm). On the other hand, lowest permeation of TPhP was determined for the smallest pore size, COF1 in which steric effects were clearly observed. The maximum adsorption capacities (qm) of TPhP were recorded in order COF2 (387.2 mg/g) > COF3 (371.2 mg/g) > COF1 (86.1 mg/g).

Despite pore size being a well-investigated, key feature of COFs materials in terms of separation and adsorption applications, other important factors such as surface charges, stability and hydrophilicity/hydrophobicity are still not deeply understood. Surface charges have been recognized as a crucial property for nanofiltration in organic solvents. This is because electrostatic interactions that contribute either to selectively attract the desired solutes or to repulse (reject) them. In contrast of metal-organic frameworks (MOFs), which have been extensively studied on the concept of charges for their functionality, this property is less studied for COFs. A series of uncharged and charged COFs were produced by Li-Oakey et al., using different functional groups [11]. They found that highly negatively charged COF’s pore using carboxylate groups (-COO^−^) produced impressive results for water flux and cation size selectivity.

In addition to being stable, the produced COF (in aqueous and organic media) must be accurately designed with covalently combined building blocks. This is a major driving force in COF research. COF-1 was successfully synthesized by condensation reaction of diboronic acid, which constituted phenyl rings linked by boroxine [12]. However, electron deficiency of boron sites in boroxine ring resulted in reduced chemical stability of COF-1, which is susceptible to nucleophilic attack. The covalent triazine-based frameworks (CTF-1) synthesized from the trimerization of nitriles in a ZnCl_2_ melt showed a stable form [13]. Since CTF-1 was produced under acidic condition, higher stability of triazine-linked rings was found owing to the lesser susceptibility of CTF-1 to hydrolytic decomposition in water and organic solvents. Incorporating COFs with different types of functional group is a demanding task in terms of attaining suitable hydrophobicity or hydrophilicity. For example, a computational study was reported for x-functionalized TpPa COFs (x = –NHCOCH_2_CH_2_CH_3_, –NHCOCH_2_-COOH, –OCH_2_C_6_H_5_, –NHCOCH_2_CH_2_NH_2_, –OCH_2_CH_2_CH_2_CH_3_, –OCH_2_CH_2_CH_2_OH and –NHCOCH_2_CH_3_) [14]. TpPa with similar aperture size experienced higher flux of pure water in hydrophilic functionalized TpPa rather than the hydrophobic functionalized ones. This was caused by preferential interactions between water and the hydrophilic groups. Additionally, the lifetime of hydrogen bond was recorded longer in hydrophilic functionalized TpPa which consequently showed higher occupancy and density of water in the pore.

Aromatic property is a key feature to explore since COFs are developed by enormous diversity of aromatic rings. Electron-rich π-system (condensed aromatic) can promote hydrogen bonding, whereas weaker aromatic surface can provide hydrophobic microenvironment for soft π-interactions [15,16]. To the best of our knowledge, however, aromaticity of COFs, as well as its relation to the stability and adsorption performance, has not been thoroughly investigated.

## 2. Computational Methods

**Figure 1:**
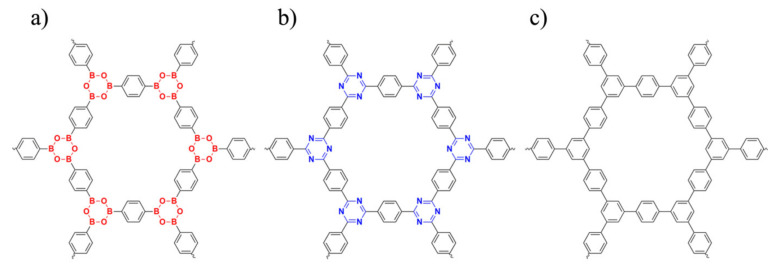
Two-dimensional molecular structure of (**a**) COF-1, (**b**) CTF-1 and (**c**) TPG that were studied in this work.

In this work, two basic COFs were selected (Figure 1), COF-1 and CTF-1, which are composed of boroxine and triazine, respectively, both of which are linked by benzene rings (the most common rings in COFs). In addition, a theoretical porous graphene (TPG) (Figure 1c), which unfortunately has not been synthesized, derived from the COFs structure is also analyzed. These three sheets were compared in terms of stability and aromatic property based on quantum mechanics (QM) study. Then, they were solvated with a 50:50 water–ethanol mixture [17] and underwent molecular dynamics (MD) simulations. MD simulations revealed the adsorption behavior of water and ethanol on the surface of COF-1, CTF-1 and TPG, which is influenced by the electronic properties, as determined from QM calculations.

### 2.1. QM Calculations

The COF-1 crystal structure (CCDC 287138) was obtained from the Cambridge Crystallographic Data Center (CCDC) and imported in GaussView 5.0 [18]. Then, all initial structures were derived and optimized by QM calculations. Hartree–Fock (HF) geometry optimization of COF-1, CTF-1 and TPG were carried out in gaseous phase using the 6-31G(d) basis set in Gaussian09 [19]. The optimized structures were utilized for frequency, NMR and natural bond orbital (NBO) calculations. Specifically, the energy gap between the highest occupied molecular orbital (HOMO) and the lowest unoccupied molecular orbital (LUMO) (E_HL_) [20], hardness (η) [21], softness (*S*) [22], point groups [23] and geometric parameters for measuring harmonic oscillator model of aromaticity (HOMA) [24] were calculated. All detailed information for measuring HOMA is available in Section SI.1.2. (Supplementary Materials). Aromatic stabilization energy (ASE) [25] was evaluated from frequency output (sum of electronic and zero-point energies) and hydrogenation enthalpy (ΔH) [26] was determined from the sum of electronic and thermal enthalpies. Nucleus-independent chemical shift (NICS) [27] was extracted from NMR calculation based on gauge-independent atomic orbital (NMR=GIAO). NBO version 3.1 [28] calculations were performed in order to find the most probable donor–acceptor interaction and its corresponding occupancy of involved atomic orbital. The NBO was analyzed by energetic examination of all possible interactions between donor NBOs (filled) and acceptor NBOs (empty), as well as computing their energetic importance by second-order perturbation theory. Hence, we were able to evaluate which interaction contributed towards the stability and electron delocalization of specific benzene ring in the three sheets studied.

### 2.2. Atomic Models and MD Simulation

Crystal structures of COF-1 and CTF-1 possess the same hcb (honeycomb) net topology since they adopt a rhombus-shaped unit crystal [29]. The crystal structures for COF-1, CTF-1 and TPG and their unit cell are illustrated in Figures S4–S6 in Supplementary Materials. All crystal structures were considered to be rigid during MD simulation, because flexibility had shown negligible effect on MD simulation results [30,31]. Universal Force Field (UFF) [32] was applied for the potential parameters of the COFs and TPG, while All-Atom Optimized Potential for Liquid Simulations (OPLS-AA) [33] and Simple Point Charge Extended (SPCE) [34] force fields were used for ethanol and water, respectively [35]. In order to compute the electrostatics interactions between liquids (ethanol and water) and the solid sheets (COF-1, CTF-1 and TPG), atomic partial charges were assigned (Section SI.1.4.). These partial charges were calculated using CHarges from Electrostatic Potentials using a Grid (CHelpG) method in Gaussian09 at B3LYP/6-31+G(d) level [36]. Each unit cell of the solid sheets was extended by 1 × 1 × 1 (super cell) and they were filled with 750 ethanol and 750 water molecules (50:50 water–ethanol mixtures).

All molecular dynamics (MD) simulations were carried out using GROMACS version 5.1.4 [37]. Firstly, energy minimization was carried out using the steepest descent minimization algorithm with a force tolerance of 10.0 kJ.mol^−1^ nm^−1^ and a step size of 0.001 nm. This was followed by canonical (constant NVT) ensemble simulation for 500 ps, the velocities were assigned by Maxwell–Boltzmann distribution at 300 K and the temperature was controlled by velocity-rescaling thermostat [38] throughout all simulations. Periodic boundary conditions were applied in all directions for all systems. Subsequently, isothermal-isobaric (constant NPT) ensemble simulation was run step-by-step for 300 ps, until the pressure stabilized at 1.0 bar using Berendsen pressure coupling [39]. Finally, the equilibrated systems underwent 50 ns MD production simulation at constant NPT ensemble. Particle Mesh Ewald (PME) method [38] was used to evaluate long-range electrostatic interactions, with a grid spacing of 0.16 nm and a fourth-order (cubic) interpolation. In addition, Coulombic and van der Waals interactions were cut off at 1.0 nm using the Verlet scheme in GROMACS. Trajectory analyses were performed for the last 20 ns of the production simulations.

## 3. Results and Discussion

**Figure 2:**
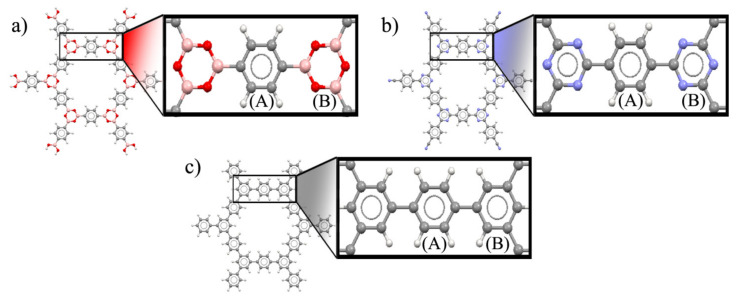
Optimized structure of (**a**) COF-1, (**b**) CTF-1 and (**c**) TPG.

The cluster structures of COF-1, CTF-1 and TPG (Figure 2) were optimized at the ground state. The dual-linked phenyl group is denoted by ring (A), whereas the three-side linked boroxine (COF-1), triazine (CTF-1) and phenyl (TPG) groups are denoted by ring (B). This is referred to initial availability of ring A in reactant structures for synthesis of COF-1 and CTF-1, while ring B is built as the secondary part [40]. All optimized structures have a D*_6h_* point group perceived mainly from one COF’s plane (σ*_h_*), one C*_6_* axis at the center of the pore perpendicular to the COF’s plane and six corresponding rotation axes of C*_2_*. Being highly symmetric (like benzene) is important for aromatic compounds because of more effective orbital overlapping and electron delocalization, leading to higher stability [41]. The aromatic properties and kinetic stability for COF-1, CTF-1 and TPG, were evaluated using nucleus-independent chemical shifts (NICS), aromatic stabilization energy (ASE), harmonic oscillator model of aromaticity (HOMA), energy difference between HOMO and LUMO (E_HL_), hardness (η) and softness (*S*) (Table 1).

**Figure 3:**
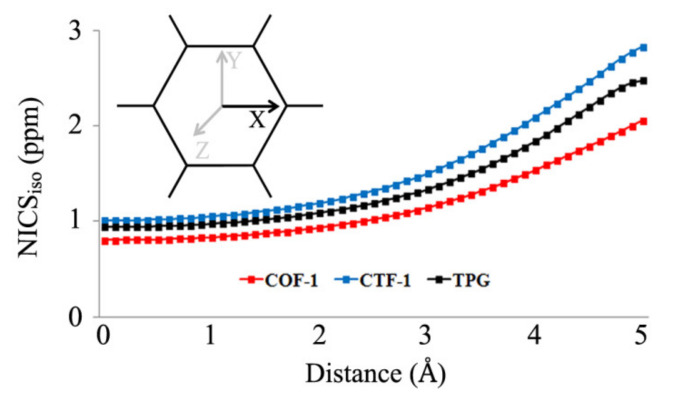
The curves of NICS_iso_ scan calculated with 0.1 Å spacing along *x*-axis.

Bq atom at center of pore showed a relatively weak paratropic current with a minor difference between NICS_zz_ value for COF-1, CTF-1 and TPG. These close values can be explained by similar pore size and distance from centre of pore to the pore wall. However, the aromaticity of rings A and B showed distinct properties. NICS_zz_ values of A rings are influenced by the neighbor rings although they are of the same type (phenyl group). The values in 1 Å above the ring plane (in bracket) are as high in the centre of ring A. According to Table 1, ring A in COF-1 has better contribution with the other two boroxine rings indicated by the lowest values, i.e., the highest diatropic current and more aromatic property. On the other side, B rings including boroxine, triazine and phenyl rings are chemically different. Among the B rings, the most aromatic current can be seen in TPG, while the most anti-aromatic current can be found in COF-1. Generally, all 12 rings are involved in constructing a pore for the structures in which the sum of aromatic property on the surface is in the order of TPG (−304.2 ppm) > CTF-1 (−203.4 ppm) > COF-1 (−19.2 ppm). With respect to the effect of aromatic property of the 12 rings joined toward building the system’s pore, the isotropic current inside the pore was explored using NICS_iso_ scan along the *x*-axis (NICS-X-scan). Bq atoms were placed with 0.1 Å spacing. The resulting NICS_iso_ values showed different curves (Figure 3). Scan of NICS_iso_ values from the center of the pore (0.0 Å) to the near pore wall (5.0 Å) indicated an upward trend for all the systems. Among them, COF-1′s pore produced the lowest values in all distances, indicating weaker anti-aromatic property (paratropic current) inside the pore. On the other hand, the NICS-X-scan curve of CTF-1 produced the highest NICS_iso_ values in all distances, implying stronger anti-aromatic property (paratropic current) within the pore.

**Figure 4:**
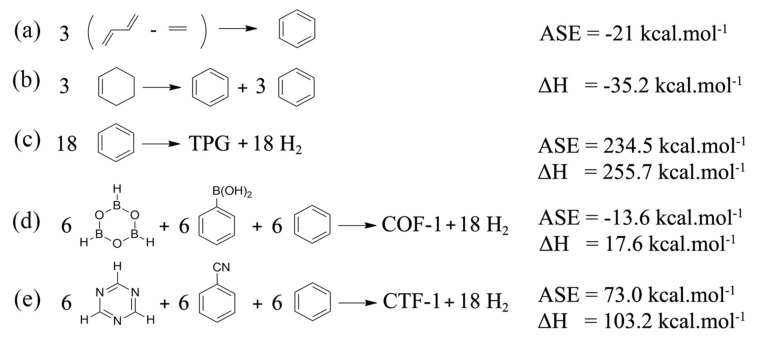
ASE and ΔH calculations for reference compounds, TPG, COF-1 and CTF-1. (**a**,**b**) ASE and ΔH calculations for the formation of benzene, respectively. (**c**–**e**) ASE and ΔH calculations for the formation of TPG, COF-1 and CTF-1, respectively.

Evaluation of aromatic property for ring A based on structural approach, HOMA, was also carried out. In this case, HOMA = 1 is shown for a complete delocalized system (benzene-like), while HOMA = 0 indicated an absolute localized system. Although here the values are not significantly differed, the closest HOMA value to 1 (0.994) in CTF-1 when compared to others implied that the phenyl rings (ring A) in CTF-1 were more influenced by the triazine rings (rings B) and it is more structurally aromatic. In aromatic systems, smaller differences between the HOMO and LUMO energy gap (E_HL_) are associated with greater aromatic character and stability. Among the structures studied, TPG has the smallest E_HL_ value (5.53 eV) which corresponds to the best electron transformation between the orbitals gap, more kinetic stability, as well as low chemical reactivity. The lowest chemical reactivity predicted for TPG is also reflected from the lowest hardness (2.75 eV) and the highest softness (0.36 eV). From the E_HL_ standpoint, COF-1 has as much electron transformation as CTF-1. Consequently, CTF-1 is predicted to possess the most localized electrons and chemical reactivity. Since COF-1, CTF-1 and TPG are a well-arranged collection of aromatic rings; aromatic stabilization energy (ASE) and hydrogenation enthalpy (ΔH) indexes can be adopted appropriately. ASE is important to discuss because it ascertains how much stability arises from aromatic current in the cyclic system when they stand under comparison with non-cyclic form. Moreover, hydrogenation enthalpy (ΔH) was also determined to understand the thermodynamic stabilization of the cyclic systems when they were built from the reactants. Following the equations of ASE (Figure 4a) and ΔH (Figure 4b) for benzene which was suggested by Schleyer et al. [42,43] for measuring the energy and enthalpy difference between reactants and products, the ones for COF-1, CTF-1 and TPG were calculated (Figure 4c–e).

The negative value of ASE and ΔH for benzene showed that the formation of benzene from the fragments led to a more stable, aromatic compound. Given ASE values (Figure 4c–e), the produced COF-1 becomes much more aromatic and stable when constructed by the corresponding fragments. Additionally, TPG has the highest positive value of ASE which means that the formation of TPG has received the least aromatic stabilization. Likewise, ΔH values suggested that constructing COF-1 required the least heat of formation and become the most thermodynamic-stabilized product when built by the relevant fragment compared to the other solid sheets. In contrast, joining 18 benzene rings (highly stable) together for producing TPG needed the most heat of formation. Figure 4c–e all showed positive ΔH values indicating endothermic reactions. All in all, both ASE and ΔH have showed that aromatic and thermodynamic stabilizations of the products are in the order of COF-1 > CTF-1 > TPG when made by their fragments.

**Figure 5:**
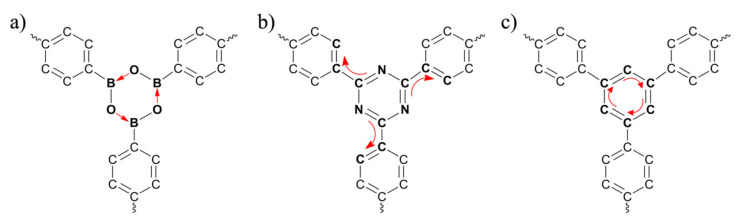
The strongest delocalization in ring B for (**a**) COF-1, (**b**) CTF-1 and (**c**) TPG.

The three investigated systems have obvious differences in the ring B position. Although the high stability and aromatic character of benzene are described by the mode of coupling the spins of the π electrons, this is not a decisive effect on boraxine and triazine (ring B) [44]. Aromatic current in nitrogen-containing rings is formed by contribution between lone pair electrons on the sp^2^-hybridized nitrogen and *p*-orbitals on carbon atoms [45]. Moreover, electron deficiency of vacant *p*-orbitals in boron can contribute to atoms having lone pair electrons as shown in borazine and boroxine molecules. However, poor delocalization of electron in boroxine is reasoned by the presence of many localized orbitals due to high electronegativity of the oxygen atoms. Therefore, NBO analysis is performed to specify the electron contribution between atoms in rings B for COF-1, CTF-1 and TPG. Donor–acceptor interactions were taken from second-order perturbation theory analysis of the NBO basis for the rings B (referred to as delocalization). Occupancy associated with that delocalization interactions are compiled in Table 2. The strongest delocalization of ring B in COF-1 showed the interaction between B–O bonding orbital of sigma-type (σ_B-O_) and unfilled valence-shell nonbonding of boron (LP*_B_) with stabilization energy of 285.4 kcal.mol^−1^. In this interaction, LP*_B_ was found to be the lowest-occupancy (0.371 electrons) and σ_B-O_ was observed to be the highest-occupancy one (1.821 electrons). Delocalization of ring B in CTF-1 is mainly influenced by the interaction between anti-bonding π orbital C=N (π*_C=N_) and anti-bonding π orbital C=C (π*_C=C_) with the stabilization energy of 177.6 kcal.mol^−1^. The difference of occupancy between the two anti-bonding orbitals is close: 0.347 and 0.349 electrons for π*_C=N_ and π*_C=C_, respectively. On the other hand, the lowest stabilization energy (44.1 kcal.mol^−1^) in ring B estimated for TPG is given by the interaction between bonding π orbital C=C (π_C=C_) and anti-bonding π orbital C=C (π*_C=C_). The corresponding interaction has the highest-occupancy orbital with 1.640 electrons and the lowest-occupancy orbital with 0.355 electrons for π_C=C_ and π*_C=C_, respectively. As a consequence, NBO analysis emphasized that ring B in COF-1 is stabilized by σ-type delocalization focused inside the ring, CTF-1 is stabilized by π-type delocalization among the rings and TPG is stabilized by π-type delocalization concentrated within the rings (red arrow depicted in Figure 5). It is noteworthy that π-type delocalization of ring B in CTF-1 has connected with rings A around.

**Figure 6:**
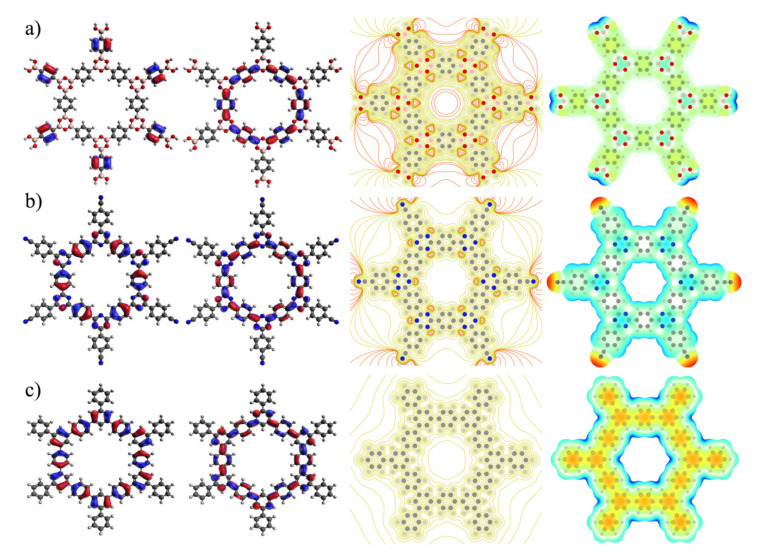
HOMO, LUMO, ESP contour and map, respectively, from left to right for (**a**) COF-1, (**b**) CTF-1 and (**c**) TPG.

Visualization of HOMO, LUMO, electrostatic potential (ESP) contour and its map are powerful practical models for the expectancy of electron density. These models were employed for the investigated COF sheets in Figure 6. HOMO orbitals covered the rings around the CTF-1′s and TPG’s pores, but there is a lack of HOMO orbitals over the rings around COF-1′s pore. However, LUMO orbitals demonstrated similar states among all surfaces. For the ESP contour, the red lines belong to negative potential, whereas green lines belong to positive potential. The ESP contour of COF-1 expressed two red circle lines in the center of the pore, as well as concentrated triangle red lines close to oxygen atoms in boroxine rings. A better side contribution between electron densities on oxygen atoms in the pore wall contributed towards the negative potential inside the pore.

This visualization supports the result of ASE for COF-1. In agreement with the ASE calculation, the ESP contour showed that electron currents inside the pore led to aromatic stabilization if COF-1 is built by those fragments (Figure 4d). Although the ESP contour of CTF-1 illustrated only a small, concentrated electron density on nitrogen atoms in triazine rings, these negative potentials did not contribute to the resulting electron density within the pore. No negative potential was detected by ESP contour for TPG since there are no lone pair electrons or any other highly concentrated electron in the structure. However, ESP map revealed that TPG sheet has provided localized electron cloud in center of phenyl ring which is not shared with neighbor rings. ESP map for COF-1 and CTF-1 has alternatively pictured a more natural electron density surface caused by higher electron delocalization. Particularly, boroxine and triazine rings (ring B) in COF-1 and CTF-1 (blue color) showed a more positive potential region or lower electron density.

**Figure 7:**
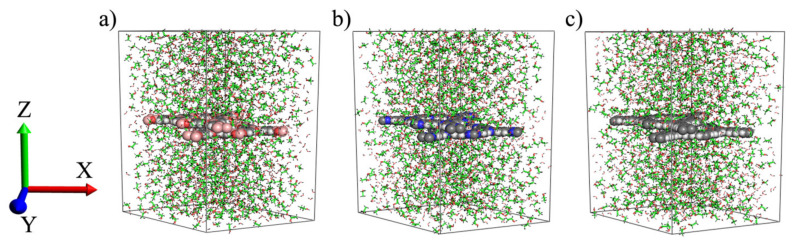
Final configuration of (**a**) COF-1, (**b**) CTF-1 and (**c**) TPG.

QM calculations of the clusters taken from COF-1, CTF-1 and TPG sheets have clearly distinguished their variety of characters. By comparison, COF-1 has presented poor aromatic current on surface and less anti-aromatic current within the pore. This is due to delocalization interaction between B–O bonding orbital of sigma type (σ_B-O_) and unfilled valence-shell nonbonding of boron (LP*_B_) in pore wall that was further illustrated by the HOMO orbitals and ESP contour. Moreover, the ASE and ΔH values showed that COF-1 is much more aromatic and thermodynamically stable when made by the corresponding fragments. CTF-1 is highlighted by the lowest softness, the highest E_HL_ and hardness that have resulted in less kinetic stability and more chemical reactivity. Moreover, TPG has shown specific properties; the strongest aromatic current on the surface, the most kinetic stability, the lowest chemical reactivity and the most localized π electron in phenyl rings, as exhibited by NBO analysis and ESP map. Besides the characters, we are also interested in detecting selective surface for sorption of 50:50 water–ethanol binary mixtures using MD simulation. First, interfacial structure properties of water–ethanol mixtures near double-layer COF-1, CTF-1 and TPG sheets were carried out. The three systems were equilibrated at 300 K and 1 bar, and their total energy values were not considerably fluctuated (Figure S7), therefore they can be considered for analysis of properties. Average total energy values are −28,003.5, −28,226.5 and −27,396.8 kJ.mol^−1^ for COF-1, CTF-1 and TPG, respectively. The final configuration of the systems is depicted in Figure 7, defined as *x*-axis perpendicular to the sheets within a complete 50:50 mixture of water–ethanol.

**Figure 8:**
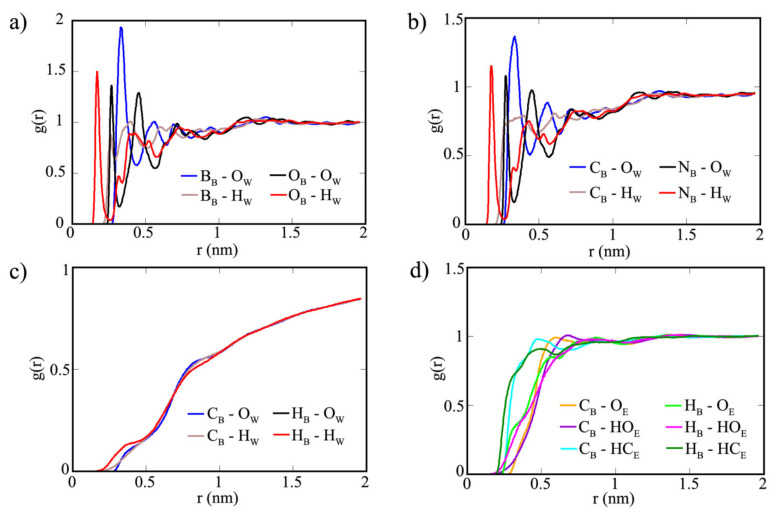
RDF graphs for (**a**) COF-1′s ring B with water, (**b**) CTF-1′s ring B with water, (**c**) TPG’s ring A with ethanol and (**d**) TPG’s ring B with ethanol.

Radial distribution function (RDF) is an appropriate descriptor that gives the probability of finding the solvent molecule near rings A and B of the sheets [46]. Additionally, RDF can be considered for characterizing the molecular interactions of the simulated systems. All RDF graphs for the three systems, the corresponding maximum pick values and their distance values are supplied in Figures S8–S10 and Table S7 in the Supplementary Materials. The surface of COF-1 was found to attract more water molecules and produced sharper peaks on ring B (Figure 8). Oxygen atoms in ring B have the closest distance (0.2 nm) with hydrogen atoms in water at the highest peak, while boron atoms in ring B are concentrated with oxygen atoms of water at a further distance. Although notable similarities are displayed between the RDFs of COF-1 and CTF-1 (Figure 8a,b), the maximum peaks are reduced in CTF-1′s ring B. Nitrogen atoms in ring B possess the strongest interaction with hydrogen atoms in water (0.2 nm) at the highest peak, while interactions of carbon atoms in ring B with oxygen atoms of water formed the highest peak at a further distance. On the other hand, all atoms in water have experienced a different condition on TPG’s surface and they were pushed away from the surface at 1.9 nm (Figure 8c). The major RDF graphs in TGP’s surface were observed for ethanol molecules. The non-polar hydrogen atoms bonded to the carbon of ethanol are the closest to the TPG’s surface (cyan and dark green lines in Figure 8d). In comparison of RDF graphs between ring A and B of TPG for ethanol, only a minor difference can be found. RDF graphs showed that surfaces in COF-1 and CTF-1 can be applicable for sorption of water through ring B, whereas surface in TPG is suitable for sorption of ethanol on ring A and B.

**Figure 9:**
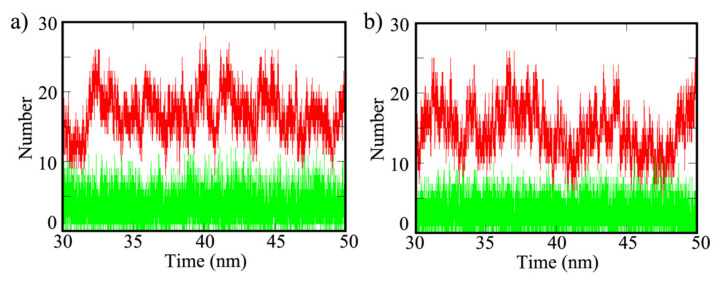
Hydrogen bond (HB) interactions of water (red) and ethanol (green) molecules on (**a**) COF-1 and (**b**) CTF-1.

To further clarify the interaction of water and ethanol on the surfaces, the hydrogen bond (HB) and non-bonded interactions were analyzed. The average number of HB interactions of water and ethanol on ring A and B for the three systems are available in Table S8 and the total values (sum of ring A and B) are plotted in Figure 9. COF-1 produced the highest HB interactions of 17.03 and 8.70 for water and ethanol, respectively, on ring B. However, there is no HB interaction shown on ring A. In comparison, the average number of HB interactions of water on CTF-1′s ring B decreased to 14.82 and ethanol on CTF-1′s ring B remained stable at 9.00. Similar to COF-1, there are no HB interactions detected on ring A for CTF-1. Both water and ethanol molecules did not form any HB interaction with TPG’s surface since there is no polar ring in structure of TPG. Figure 9 indicates the accessibility of water and ethanol molecules to HB interaction over COF-1 and CTF-1. The higher polarity of water resulted to more HB interactions than ethanol. Interestingly, the diagrams of water have considerably fluctuated over the simulation time, while the diagrams of ethanol have a levelling-off period. This phenomenon can be ascribed by the smaller size and quicker dynamical motion of water molecules, as well as the steady motion of ethanol due to heavier molecular weight.

Non-bonded interactions are weaker than HB interaction and they are divided into van der Waals (vdw) and electrostatic (Es) interactions [47,48]. Van der Waals interaction is responsible for attraction and repulsive forces due to permanent electric dipoles in particles (surface electrons) and it is defined by a Lennard–Jones potential. Electrostatic interaction arises from unequal distribution of charges between particles and it is modeled by a coulomb potential. As can be seen in Table 3, vdw and Es interaction energies have further elucidated the material’s surface for interacting with water or ethanol. In a 50:50 mixture of water–ethanol, ethanol molecules have recorded the lowest vdw interaction energy value (−1165.1 kJ.mol^−1^) on TPG and the highest Es interaction energy value (−26.3 kJ.mol^−1^) on TPG. This means ethanol molecules have mostly interacted on TPG’s surface by non-polar hydrogen atoms via vdw forces and this is in agreement with the RDF analysis. In contrast, the interaction of water molecules on the surface of COF-1 and CTF-1 is mainly via Es interaction with the lowest energy of −915.4 and −640.2 kJ.mol^−1^, respectively. The highest energy was also observed for Es energy of water on TPG’s surface (−7.4 kJ.mol^−1^). As a result, water molecules preferred to be adsorbed on COF-1 and CTF-1′s surfaces via electrostatic (Es) interaction which is governed by distribution of charges.

**Figure 10:**
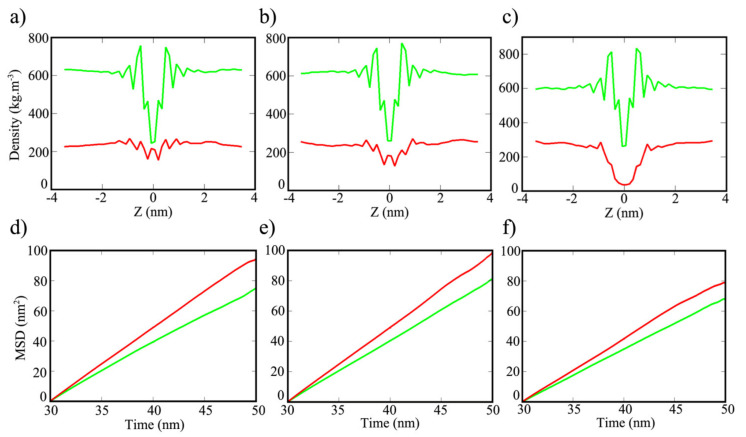
Density profiles of the center of mass for water (red lines) and ethanol (green lines) on (**a**) COF-1, (**b**) CTF-1 and (**c**) TPG, and MSD diagrams of the ethanol (green lines) and water (red lines) molecules on (**d**) COF-1, (**e**) CTF-1 and (**f**) TPG.

Valuable feedback on the study of interactions can be given in terms of partial density and diffusion of the solvents on the three surfaces. Water molecules are strongly packed together with less space between molecules when compared to the packed ethanol molecules at pure condition, thus water has higher density [17,49]. Nevertheless, partial density in the center of mass for 50:50 mixture of water–ethanol on COF-1, CTF-1 and TPG displayed that the interfacial water densities are less than ethanol at all over the distances (Figure 10a–c). The maximum and minimum values of the corresponding densities are arranged in Table S9. The minimum density of water on COF-1 was 153 kg.m^−3^ at 0.21 nm from center of the sheet, and it is slightly reduced to 127 kg.m^−3^ at the same distance. Moreover, the density of water molecules dropped to the lowest value of 35 kg.m^−3^ at 0.07 nm on TPG’s surface. The density of ethanol that was higher than water on the three sheets showed a maximum value of 756 kg.m^−3^ at 0.49 nm from the center of the COF-1′s surface. Within the same distance, the maximum density of ethanol increased to 769 and 832 kg.m^−3^ for CTF-1 and TPG, respectively. According to the graphs, the closest difference between water and ethanol can be observed at the center of the COF’s sheet, while the largest difference can be found at the center of TPG’s sheet. Estimation of mean-squared displacement (MSD) described the mobility of ethanol and water molecules over the surface of COF-1, CTF-1 and TPG. According to the diagrams in Figure 10d–f, higher values (more mobility) of water are clearly visible over time which can justify the violent fluctuation of HB interaction. MSD values are relatively close for COF-1 and CTF-1 (Table S10). The MSD values of ethanol and water molecules were 0.61 and 0.80 (10^−5^ cm^2^ s^−1^), respectively, on COF-1′s surface, whereas the MSD values of ethanol and water molecules were 0.67 and 0.82 (10^−5^ cm^2^ s^−1^), respectively on CTF-1′s surface. However, the mobility of ethanol and water molecules was much slower in TPG, and the MSD values were reduced to 0.57 and 0.69 (10^−5^ cm^2^ s^−1^) for ethanol and water, respectively.

Overall, MD simulations have imparted valuable information about the adsorption of water and ethanol molecules on the surface of COF-1, CTF-1 and TPG. Trajectory analysis has demonstrated that COF-1 and CTF-1 structure behave similarly against the dynamic behavior of water and ethanol molecules. Both appeared as a suitable platform for water adsorption (on rings B) as shown in RDF analysis. Prominent water adsorption on COF-1 and CTF-1 was further revealed by hydrogen bond (HB) and electrostatic (Es) non-bonded interaction. Additionally, the interfacial water on COF-1 and CTF-1′s surface had higher density and mobility than on TPG’s surface. In contrast, TPG has variously performed as a sorbent sheet inside 50:50 mixture of water–ethanol. For instance, the surface of TPG produced a considerable amount of RDF peaks for non-polar hydrogen atoms of ethanol molecules. Furthermore, adsorption of ethanol on the surface of TPG is mainly governed by van der Waals interactions. Higher density and slower mobility of ethanol was observed on the surface of TPG. The thermodynamics of solvent adsorption on COFs can be explain using the Flory–Huggins theory for two-phase systems [50,51]. This should be one of the focus of further studies of these systems.

## 4. Conclusions

In this study, three solid sheets including COF-1, CTF-1 and TPG were firstly explored for aromatic and stability characters using QM calculations. Next, they were soaked in 50:50 mixture of water–ethanol in order to find their potency for selective adsorption by means of MD simulation. COF-1 showed poor aromatic properties due to main delocalization interaction between B-O bonding orbital of sigma type (σ_B-O_) and unfilled valence-shell nonbonding of boron (LP*_B_). It becomes the most aromatic and thermodynamically stable when built by its own fragments, since a considerable electron current is created in COF-1′s pore. CTF-1 was demonstrated to be the least kinetically stable and the most chemically reactive compound. Both COF-1 and CTF-1 showed good potential for surface adsorption of water molecules by hydrogen bond (HB) and electrostatic (Es) interactions. On the other hand, TPG is determined as the most aromatic, having the most localized π electrons on phenyl rings, and being the least chemical reactive. The character of TPG’s surface was reflected by MD simulation as the most appropriate solid sheet for the adsorption of ethanol through van der Waals interaction with non-polar hydrogen or ethanol molecules. We have shown through computational analysis that COFs materials have the potential as solid sheets for selective adsorption of polar solvents, since the rings formed, such as boroxine and triazine, have reduced aromatic current and π electron cloud on the surfaces.

## Figures and Tables

**Table 1:** Aromaticity and stability indexes for three surfaces.

Properties	COF-1	CTF-1	TPG
NICS_zz_(pore) ^1^	3.9	4.6	4.3
NICS_zz_(A)	−10.5 [−28.4]	−7.2 [−25.3]	−5.3 [−23.7]
NICS_zz_(B)	30.5 [5.2]	12.2 [−13.6]	−0.8 [−20.9]
NICS_zz_(tot)	−19.2	−203.4	−304.2
HOMA	0.989	0.994	0.990
HOMO (eV)	−8.70	−9.49	−7.34
LUMO (eV)	2.00	0.33	1.81
E_HL_ (eV)	6.70	9.16	5.53
η (eV)	3.35	4.57	2.75
*S* (eV)	0.29	0.21	0.36

^1^ The unit for NICS value is ppm.

**Table 2:** Second-order perturbation theory analysis and occupancy of natural orbitals (NBOs).

Properties	COF-1	CTF-1	TPG
Donor → acceptor	σ_B-O_ → LP*_B_	Π *_C=N_ → π*_C=C_	π_C=C_ → π*_C=C_
*E_ij_* (kcal.mol^−1^)	285.4	177.6	44.1
Occupancy of σ_B-O_	1.821	-	-
Occupancy of LP*_B_	0.371	-	-
Occupancy of π*_C=N_	-	0.347	-
Occupancy of π*_C=C_	-	0.349	-
Occupancy of π_C=C_	-	-	1.640
Occupancy of π*_C=C_	-	-	0.355

**Table 3:** Non-bonded interaction energies values.

Solvent		COF-1	CTF-1	TPG
Ethanol (kJ.mol^−1^)	vdw	−985.0	−906.5	−1165.1
	Es	−381.1	−302.1	−26.3
Water (kJ.mol^−1^)	vdw	−37.7	−29.7	−59.6
	Es	−915.4	−640.2	−7.4

## Data Availability

The data presented in this study are available on request from the corresponding author.

## Supplementary Materials

All materials are included in the Supplementary Materials file, available online at https://www.mdpi.com/article/10.3390/polym13111861/s1.
