# Supplementary material for: Elucidating the Aromatic Properties of Covalent Organic Frameworks Surface for Enhanced Polar Solvent Adsorption
Source: Polymers (Basel). 2021 Jun 3;13(11):1861. doi: 10.3390/polym13111861 (PMC8199954; doi:10.3390/polym13111861)
Supplement: Supplementary file 1 [file polymers-13-01861-s001.zip › polymers-1221307-supplementary.pdf]

Supplementary information

Elucidating the Aromatic Properties of Covalent Organic Frameworks Surface for Enhanced Polar Solvent Adsorption

Mostafa Yousefzadeh Borzehandani[1,2], Emilia Abdulmalek[1,2], Mohd Basyaruddin Abdul Rahman[1,2] and Muhammad Alif Mohammad Latif[1,3] *

[1] *Integrated Chemical BioPhysics Research, Faculty of Science, Universiti Putra Malaysia, 43400 UPM Serdang, Selangor, MALAYSIA*

[2] *Department of Chemistry, Faculty of Science, Universiti Putra Malaysia, 43400 UPM Serdang, Selangor, MALAYSIA*

[3] *Centre of Foundation Studies for Agricultural Science, Universiti Putra Malaysia, 43400 UPM Serdang, Selangor, MALAYSIA*

*Corresponding author email address: aliflatif@upm.edu.my

Contents

SI.1. QM calculation
SI.1.1. Optimization and frequency calculations …………………………………………………… S2
SI.1.2. HOMA parameters ……………………………………………………………………… S17
SI.1.3. NICS-X-scan ……………………………………………………………………………... S18
SI.1.4. Atomic charges ………………………………………………………………………….. S20

SI.2. MD simulation
SI.2.1. Super cell and unit cell …………………………………………………………………. S22
SI.2.2. Potential parameters ……………………………………………………………………. S24
SI.2.3. Total energy ……………………………………………………………………………. S25
SI.2.4. Radial distribution function (RDF) ……………………………………………………. S26
SI.2.5. Hydrogen bond (HB) interaction ………………………………………………………. S30
SI.2.6. Partial density …………………………………………………………………………. S31
SI.2.7. Mean square displacement (MSD) ……………………………………………………. S31
References …………………………………………………………………………………… S32

SI.1.1. Optimization and frequency calculation

Sum of electronic and zero-point energies (hartree) used for ASE, sum of electronic and thermal enthalpies (hartree) applied for ΔH equations, and x, y, z coordinates (Å) for the optimized structures [1]:

COF-1

$E_0 = E_{elec} + ZPE$ = -5601.440178

$H = E + RT$ = -5601.334262

Center Number	Atomic Number	Atomic Type	Coordinates (Angstroms) X	Y	Z
1	5	0	5.041166	6.341328	0.000000
2	5	0	8.012334	1.195113	0.000000
3	8	0	5.707193	7.535776	0.000000
4	8	0	9.379770	1.174686	0.000000
5	6	0	7.909599	3.768048	0.000000
6	6	0	7.229811	2.549671	0.000000
7	6	0	7.218025	4.965889	0.000000
8	6	0	5.822985	4.986364	0.000000
9	1	0	8.984512	3.773308	0.000000
10	1	0	7.760036	5.894161	0.000000
11	5	0	2.971169	7.536442	0.000000
12	8	0	3.672396	6.360777	0.000000
13	6	0	1.406826	7.536035	0.000000
14	6	0	0.691551	6.338153	0.000000
15	6	0	-0.691551	6.338153	0.000000
16	6	0	-1.406826	7.536035	0.000000
17	1	0	1.224311	5.404525	0.000000
18	5	0	-2.971169	7.536442	0.000000
19	8	0	-3.672396	6.360777	0.000000
20	1	0	-1.224311	5.404525	0.000000
21	5	0	5.042358	8.733620	0.000000
22	8	0	3.672577	8.710462	0.000000
23	6	0	5.823110	10.085922	0.000000
24	6	0	5.144694	11.305109	0.000000
25	6	0	5.836455	12.503063	0.000000
26	6	0	7.231126	12.524677	0.000000
27	1	0	4.069579	11.311570	0.000000
28	5	0	8.017075	13.885981	0.000000
29	1	0	5.294405	13.431243	0.000000
30	6	0	5.143226	3.767977	0.000000
31	6	0	5.834777	2.570176	0.000000
32	1	0	4.068300	3.762547	0.000000
33	8	0	7.344793	0.000000	0.000000
34	1	0	5.292611	1.641978	0.000000
35	6	0	7.218164	10.107990	0.000000
36	6	0	7.909742	11.306050	0.000000
37	1	0	7.761317	9.180144	0.000000

38	1	0	8.984595	11.300711	0.000000
39	6	0	0.691574	8.733937	0.000000
40	6	0	-0.691574	8.733937	0.000000
41	1	0	1.224475	9.667469	0.000000
42	8	0	-3.672577	8.710462	0.000000
43	1	0	-1.224475	9.667469	0.000000
44	5	0	-8.017075	13.885981	0.000000
45	6	0	-7.231126	12.524677	0.000000
46	6	0	-5.836455	12.503063	0.000000
47	6	0	-5.144694	11.305109	0.000000
48	6	0	-5.823110	10.085922	0.000000
49	1	0	-5.294405	13.431243	0.000000
50	5	0	-5.042358	8.733620	0.000000
51	1	0	-4.069579	11.311570	0.000000
52	6	0	-7.909742	11.306050	0.000000
53	6	0	-7.218164	10.107990	0.000000
54	1	0	-8.984595	11.300711	0.000000
55	8	0	-5.707193	7.535776	0.000000
56	1	0	-7.761317	9.180144	0.000000
57	5	0	10.084715	0.000000	0.000000
58	5	0	-5.041166	6.341328	0.000000
59	5	0	8.012334	-1.195113	0.000000
60	5	0	5.042358	-8.733620	0.000000
61	5	0	8.017075	-13.885981	0.000000
62	8	0	5.707193	-7.535776	0.000000
63	6	0	7.909742	-11.306050	0.000000
64	6	0	7.231126	-12.524677	0.000000
65	6	0	7.218164	-10.107990	0.000000
66	6	0	5.823110	-10.085922	0.000000
67	1	0	8.984595	-11.300711	0.000000
68	1	0	7.761317	-9.180144	0.000000
69	5	0	2.971169	-7.536442	0.000000
70	8	0	3.672577	-8.710462	0.000000
71	6	0	1.406826	-7.536035	0.000000
72	6	0	0.691574	-8.733937	0.000000
73	6	0	-0.691574	-8.733937	0.000000
74	6	0	-1.406826	-7.536035	0.000000
75	1	0	1.224475	-9.667469	0.000000
76	5	0	-2.971169	-7.536442	0.000000
77	8	0	-3.672577	-8.710462	0.000000
78	1	0	-1.224475	-9.667469	0.000000
79	5	0	5.041166	-6.341328	0.000000
80	8	0	3.672396	-6.360777	0.000000
81	6	0	5.822985	-4.986364	0.000000
82	6	0	5.143226	-3.767977	0.000000
83	6	0	5.834777	-2.570176	0.000000
84	6	0	7.229811	-2.549671	0.000000
85	1	0	4.068300	-3.762547	0.000000
86	1	0	5.292611	-1.641978	0.000000
87	6	0	5.144694	-11.305109	0.000000

88	6	0	5.836455	-12.503063	0.000000
89	1	0	4.069579	-11.311570	0.000000
90	1	0	5.294405	-13.431243	0.000000
91	6	0	7.218025	-4.965889	0.000000
92	6	0	7.909599	-3.768048	0.000000
93	1	0	7.760036	-5.894161	0.000000
94	8	0	9.379770	-1.174686	0.000000
95	1	0	8.984512	-3.773308	0.000000
96	6	0	0.691551	-6.338153	0.000000
97	6	0	-0.691551	-6.338153	0.000000
98	1	0	1.224311	-5.404525	0.000000
99	8	0	-3.672396	-6.360777	0.000000
100	1	0	-1.224311	-5.404525	0.000000
101	5	0	-10.084715	0.000000	0.000000
102	5	0	-16.034149	0.000000	0.000000
103	8	0	-9.379770	-1.174686	0.000000
104	6	0	-13.746198	-1.197013	0.000000
105	6	0	-14.462252	0.000000	0.000000
106	6	0	-12.362859	-1.197118	0.000000
107	6	0	-11.646220	0.000000	0.000000
108	1	0	-14.279000	-2.130532	0.000000
109	1	0	-11.830896	-2.131426	0.000000
110	5	0	-8.012334	1.195113	0.000000
111	8	0	-9.379770	1.174686	0.000000
112	6	0	-7.229811	2.549671	0.000000
113	6	0	-7.909599	3.768048	0.000000
114	6	0	-7.218025	4.965889	0.000000
115	6	0	-5.822985	4.986364	0.000000
116	1	0	-8.984512	3.773308	0.000000
117	1	0	-7.760036	5.894161	0.000000
118	5	0	-8.012334	-1.195113	0.000000
119	8	0	-7.344793	0.000000	0.000000
120	6	0	-7.229811	-2.549671	0.000000
121	6	0	-5.834777	-2.570176	0.000000
122	6	0	-5.143226	-3.767977	0.000000
123	6	0	-5.822985	-4.986364	0.000000
124	1	0	-5.292611	-1.641978	0.000000
125	5	0	-5.041166	-6.341328	0.000000
126	1	0	-4.068300	-3.762547	0.000000
127	6	0	-12.362859	1.197118	0.000000
128	6	0	-13.746198	1.197013	0.000000
129	1	0	-11.830896	2.131426	0.000000
130	1	0	-14.279000	2.130532	0.000000
131	6	0	-7.909599	-3.768048	0.000000
132	6	0	-7.218025	-4.965889	0.000000
133	1	0	-8.984512	-3.773308	0.000000
134	8	0	-5.707193	-7.535776	0.000000
135	1	0	-7.760036	-5.894161	0.000000
136	6	0	-5.834777	2.570176	0.000000
137	6	0	-5.143226	3.767977	0.000000

138	1	0	-5.292611	1.641978	0.000000
139	1	0	-4.068300	3.762547	0.000000
140	5	0	-5.042358	-8.733620	0.000000
141	5	0	-8.017075	-13.885981	0.000000
142	6	0	-7.231126	-12.524677	0.000000
143	6	0	-7.909742	-11.306050	0.000000
144	6	0	-7.218164	-10.107990	0.000000
145	6	0	-5.823110	-10.085922	0.000000
146	1	0	-8.984595	-11.300711	0.000000
147	1	0	-7.761317	-9.180144	0.000000
148	6	0	-5.836455	-12.503063	0.000000
149	6	0	-5.144694	-11.305109	0.000000
150	1	0	-5.294405	-13.431243	0.000000
151	1	0	-4.069579	-11.311570	0.000000
152	5	0	16.034149	0.000000	0.000000
153	6	0	14.462252	0.000000	0.000000
154	6	0	13.746198	-1.197013	0.000000
155	6	0	12.362859	-1.197118	0.000000
156	6	0	11.646220	0.000000	0.000000
157	1	0	14.279000	-2.130532	0.000000
158	1	0	11.830896	-2.131426	0.000000
159	6	0	13.746198	1.197013	0.000000
160	6	0	12.362859	1.197118	0.000000
161	1	0	14.279000	2.130532	0.000000
162	1	0	11.830896	2.131426	0.000000
163	8	0	9.378312	-13.850300	0.000000
164	1	0	9.822000	-14.685439	0.000000
165	8	0	7.305556	-15.047007	0.000000
166	1	0	7.806963	-15.848821	0.000000
167	8	0	-7.305556	-15.047007	0.000000
168	1	0	-7.806963	-15.848821	0.000000
169	8	0	-9.378312	-13.850300	0.000000
170	1	0	-9.822000	-14.685439	0.000000
171	8	0	-16.683868	-1.196706	0.000000
172	1	0	-17.628963	-1.163382	0.000000
173	8	0	-16.683868	1.196706	0.000000
174	1	0	-17.628963	1.163382	0.000000
175	8	0	-9.378312	13.850300	0.000000
176	1	0	-9.822000	14.685439	0.000000
177	8	0	-7.305556	15.047007	0.000000
178	1	0	-7.806963	15.848821	0.000000
179	8	0	7.305556	15.047007	0.000000
180	1	0	7.806963	15.848821	0.000000
181	8	0	9.378312	13.850300	0.000000
182	1	0	9.822000	14.685439	0.000000
183	8	0	16.683868	1.196706	0.000000
184	1	0	17.628963	1.163382	0.000000
185	8	0	16.683868	-1.196706	0.000000
186	1	0	17.628963	-1.163382	0.000000

CTF-1

$E_0 = E_{elec} + ZPE = -4969.104956$

$H = E + RT = -4969.01747$

Center Number	Atomic Number	Atomic Type	Coordinates (Angstroms) X	Y	Z
1	6	0	7.647824	3.618151	0.000000
2	6	0	6.960545	2.408707	0.000000
3	6	0	6.957322	4.814135	0.000000
4	6	0	5.566274	4.823655	0.000000
5	1	0	8.719340	3.617569	0.000000
6	1	0	7.492577	5.742385	0.000000
7	6	0	1.394271	7.232362	0.000000
8	6	0	0.690510	6.032330	0.000000
9	6	0	-0.690510	6.032330	0.000000
10	6	0	-1.394271	7.232362	0.000000
11	1	0	1.227354	5.104957	0.000000
12	1	0	-1.227354	5.104957	0.000000
13	6	0	5.565843	9.640322	0.000000
14	6	0	4.875068	10.848149	0.000000
15	6	0	5.562510	12.045973	0.000000
16	6	0	6.952111	12.041409	0.000000
17	1	0	3.803790	10.844194	0.000000
18	1	0	5.029959	12.978307	0.000000
19	6	0	4.878896	3.614164	0.000000
20	6	0	5.569405	2.418166	0.000000
21	1	0	3.807345	3.615399	0.000000
22	1	0	5.034700	1.489559	0.000000
23	6	0	6.957238	9.646007	0.000000
24	6	0	7.650864	10.840262	0.000000
25	1	0	7.489452	8.716276	0.000000
26	1	0	8.724564	10.845225	0.000000
27	6	0	0.690502	8.432285	0.000000
28	6	0	-0.690502	8.432285	0.000000
29	1	0	1.226763	9.359955	0.000000
30	1	0	-1.226763	9.359955	0.000000
31	6	0	-6.952111	12.041409	0.000000
32	6	0	-5.562510	12.045973	0.000000
33	6	0	-4.875068	10.848149	0.000000
34	6	0	-5.565843	9.640322	0.000000
35	1	0	-5.029959	12.978307	0.000000
36	1	0	-3.803790	10.844194	0.000000
37	6	0	-7.650864	10.840262	0.000000
38	6	0	-6.957238	9.646007	0.000000
39	1	0	-8.724564	10.845225	0.000000
40	1	0	-7.489452	8.716276	0.000000
41	6	0	7.650864	-10.840262	0.000000

42	6	0	6.952111	-12.041409	0.000000
43	6	0	6.957238	-9.646007	0.000000
44	6	0	5.565843	-9.640322	0.000000
45	1	0	8.724564	-10.845225	0.000000
46	1	0	7.489452	-8.716276	0.000000
47	6	0	1.394271	-7.232362	0.000000
48	6	0	0.690502	-8.432285	0.000000
49	6	0	-0.690502	-8.432285	0.000000
50	6	0	-1.394271	-7.232362	0.000000
51	1	0	1.226763	-9.359955	0.000000
52	1	0	-1.226763	-9.359955	0.000000
53	6	0	5.566274	-4.823655	0.000000
54	6	0	4.878896	-3.614164	0.000000
55	6	0	5.569405	-2.418166	0.000000
56	6	0	6.960545	-2.408707	0.000000
57	1	0	3.807345	-3.615399	0.000000
58	1	0	5.034700	-1.489559	0.000000
59	6	0	4.875068	-10.848149	0.000000
60	6	0	5.562510	-12.045973	0.000000
61	1	0	3.803790	-10.844194	0.000000
62	1	0	5.029959	-12.978307	0.000000
63	6	0	6.957322	-4.814135	0.000000
64	6	0	7.647824	-3.618151	0.000000
65	1	0	7.492577	-5.742385	0.000000
66	1	0	8.719340	-3.617569	0.000000
67	6	0	0.690510	-6.032330	0.000000
68	6	0	-0.690510	-6.032330	0.000000
69	1	0	1.227354	-5.104957	0.000000
70	1	0	-1.227354	-5.104957	0.000000
71	6	0	-13.213374	-1.205712	0.000000
72	6	0	-13.904221	0.000000	0.000000
73	6	0	-11.832306	-1.202141	0.000000
74	6	0	-11.131685	0.000000	0.000000
75	1	0	-13.754523	-2.133081	0.000000
76	1	0	-11.293242	-2.127918	0.000000
77	6	0	-6.960545	2.408707	0.000000
78	6	0	-7.647824	3.618151	0.000000
79	6	0	-6.957322	4.814135	0.000000
80	6	0	-5.566274	4.823655	0.000000
81	1	0	-8.719340	3.617569	0.000000
82	1	0	-7.492577	5.742385	0.000000
83	6	0	-6.960545	-2.408707	0.000000
84	6	0	-5.569405	-2.418166	0.000000
85	6	0	-4.878896	-3.614164	0.000000
86	6	0	-5.566274	-4.823655	0.000000
87	1	0	-5.034700	-1.489559	0.000000
88	1	0	-3.807345	-3.615399	0.000000
89	6	0	-11.832306	1.202141	0.000000
90	6	0	-13.213374	1.205712	0.000000
91	1	0	-11.293242	2.127918	0.000000

92	1	0	-13.754523	2.133081	0.000000
93	6	0	-7.647824	-3.618151	0.000000
94	6	0	-6.957322	-4.814135	0.000000
95	1	0	-8.719340	-3.617569	0.000000
96	1	0	-7.492577	-5.742385	0.000000
97	6	0	-5.569405	2.418166	0.000000
98	6	0	-4.878896	3.614164	0.000000
99	1	0	-5.034700	1.489559	0.000000
100	1	0	-3.807345	3.615399	0.000000
101	6	0	-6.952111	-12.041409	0.000000
102	6	0	-7.650864	-10.840262	0.000000
103	6	0	-6.957238	-9.646007	0.000000
104	6	0	-5.565843	-9.640322	0.000000
105	1	0	-8.724564	-10.845225	0.000000
106	1	0	-7.489452	-8.716276	0.000000
107	6	0	-5.562510	-12.045973	0.000000
108	6	0	-4.875068	-10.848149	0.000000
109	1	0	-5.029959	-12.978307	0.000000
110	1	0	-3.803790	-10.844194	0.000000
111	6	0	13.904221	0.000000	0.000000
112	6	0	13.213374	-1.205712	0.000000
113	6	0	11.832306	-1.202141	0.000000
114	6	0	11.131685	0.000000	0.000000
115	1	0	13.754523	-2.133081	0.000000
116	1	0	11.293242	-2.127918	0.000000
117	6	0	13.213374	1.205712	0.000000
118	6	0	11.832306	1.202141	0.000000
119	1	0	13.754523	2.133081	0.000000
120	1	0	11.293242	2.127918	0.000000
121	6	0	4.821473	-8.351036	0.000000
122	6	0	4.822771	-6.111350	0.000000
123	6	0	2.881199	-7.232317	0.000000
124	6	0	-4.821473	-8.351036	0.000000
125	6	0	-2.881199	-7.232317	0.000000
126	6	0	-4.822771	-6.111350	0.000000
127	6	0	7.703970	-1.120967	0.000000
128	6	0	9.642946	0.000000	0.000000
129	6	0	7.703970	1.120967	0.000000
130	6	0	4.822771	6.111350	0.000000
131	6	0	4.821473	8.351036	0.000000
132	6	0	2.881199	7.232317	0.000000
133	6	0	-2.881199	7.232317	0.000000
134	6	0	-4.821473	8.351036	0.000000
135	6	0	-4.822771	6.111350	0.000000
136	6	0	-7.703970	1.120967	0.000000
137	6	0	-7.703970	-1.120967	0.000000
138	6	0	-9.642946	0.000000	0.000000
139	7	0	-3.502130	-8.399558	0.000000
140	7	0	-5.523166	-7.232713	0.000000
141	7	0	-3.502328	-6.066211	0.000000

142	7	0	3.502130	-8.399558	0.000000
143	7	0	5.523166	-7.232713	0.000000
144	7	0	3.502328	-6.066211	0.000000
145	7	0	9.025296	-1.166846	0.000000
146	7	0	9.025296	1.166846	0.000000
147	7	0	7.004657	0.000000	0.000000
148	7	0	5.523166	7.232713	0.000000
149	7	0	3.502328	6.066211	0.000000
150	7	0	3.502130	8.399558	0.000000
151	7	0	-3.502328	6.066211	0.000000
152	7	0	-3.502130	8.399558	0.000000
153	7	0	-5.523166	7.232713	0.000000
154	7	0	-7.004657	0.000000	0.000000
155	7	0	-9.025296	-1.166846	0.000000
156	7	0	-9.025296	1.166846	0.000000
157	6	0	7.674807	-13.293155	0.000000
158	7	0	8.242850	-14.277035	0.000000
159	6	0	15.349613	0.000000	0.000000
160	7	0	16.485700	0.000000	0.000000
161	6	0	7.674807	13.293155	0.000000
162	7	0	8.242850	14.277035	0.000000
163	6	0	-7.674807	13.293155	0.000000
164	7	0	-8.242850	14.277035	0.000000
165	6	0	-15.349613	0.000000	0.000000
166	7	0	-16.485700	0.000000	0.000000
167	6	0	-7.674807	-13.293155	0.000000
168	7	0	-8.242850	-14.277035	0.000000

TPG

$E_0 = E_{elec} + ZPE$ = -4130.252253

$H = E + RT$ = -4130.187133

Center Number	Atomic Number	Atomic Type	Coordinates (Angstroms) X	Y	Z
1	6	0	7.877435	3.750554	0.000000
2	6	0	7.228035	2.517357	0.000000
3	6	0	7.186792	4.946782	0.000000
4	6	0	5.794113	5.000984	0.000000
5	1	0	8.947591	3.808568	0.000000
6	1	0	7.772112	5.844557	0.000000
7	6	0	1.433922	7.518341	0.000000
8	6	0	0.690597	6.339367	0.000000
9	6	0	-0.690597	6.339367	0.000000
10	6	0	-1.433922	7.518341	0.000000
11	1	0	1.175521	5.383610	0.000000
12	1	0	-1.175521	5.383610	0.000000
13	6	0	5.796430	10.039711	0.000000
14	6	0	5.133866	11.268822	0.000000
15	6	0	5.823921	12.468244	0.000000
16	1	0	4.063301	11.315090	0.000000
17	1	0	5.273255	13.392062	0.000000
18	6	0	5.144754	3.767758	0.000000
19	6	0	5.835351	2.571609	0.000000
20	1	0	4.074582	3.709836	0.000000
21	1	0	5.250103	1.673774	0.000000
22	6	0	7.192153	10.080470	0.000000
23	6	0	7.885855	11.277786	0.000000
24	1	0	7.767505	9.176467	0.000000
25	1	0	8.961239	11.262804	0.000000
26	6	0	0.690642	8.697335	0.000000
27	6	0	-0.690642	8.697335	0.000000
28	1	0	1.175479	9.653125	0.000000
29	1	0	-1.175479	9.653125	0.000000
30	6	0	-5.823921	12.468244	0.000000
31	6	0	-5.133866	11.268822	0.000000
32	6	0	-5.796430	10.039711	0.000000
33	1	0	-5.273255	13.392062	0.000000
34	1	0	-4.063301	11.315090	0.000000
35	6	0	-7.885855	11.277786	0.000000
36	6	0	-7.192153	10.080470	0.000000
37	1	0	-8.961239	11.262804	0.000000
38	1	0	-7.767505	9.176467	0.000000
39	6	0	7.885855	-11.277786	0.000000
40	6	0	7.192153	-10.080470	0.000000
41	6	0	5.796430	-10.039711	0.000000

42	1	0	8.961239	-11.262804	0.000000
43	1	0	7.767505	-9.176467	0.000000
44	6	0	1.433922	-7.518341	0.000000
45	6	0	0.690642	-8.697335	0.000000
46	6	0	-0.690642	-8.697335	0.000000
47	6	0	-1.433922	-7.518341	0.000000
48	1	0	1.175479	-9.653125	0.000000
49	1	0	-1.175479	-9.653125	0.000000
50	6	0	5.794113	-5.000984	0.000000
51	6	0	5.144754	-3.767758	0.000000
52	6	0	5.835351	-2.571609	0.000000
53	6	0	7.228035	-2.517357	0.000000
54	1	0	4.074582	-3.709836	0.000000
55	1	0	5.250103	-1.673774	0.000000
56	6	0	5.133866	-11.268822	0.000000
57	6	0	5.823921	-12.468244	0.000000
58	1	0	4.063301	-11.315090	0.000000
59	1	0	5.273255	-13.392062	0.000000
60	6	0	7.186792	-4.946782	0.000000
61	6	0	7.877435	-3.750554	0.000000
62	1	0	7.772112	-5.844557	0.000000
63	1	0	8.947591	-3.808568	0.000000
64	6	0	0.690597	-6.339367	0.000000
65	6	0	-0.690597	-6.339367	0.000000
66	1	0	1.175521	-5.383610	0.000000
67	1	0	-1.175521	-5.383610	0.000000
68	6	0	-13.709776	-1.190458	0.000000
69	6	0	-12.326019	-1.188353	0.000000
70	6	0	-11.592859	0.000000	0.000000
71	1	0	-14.234494	-2.129258	0.000000
72	1	0	-11.830806	-2.138623	0.000000
73	6	0	-7.228035	2.517357	0.000000
74	6	0	-7.877435	3.750554	0.000000
75	6	0	-7.186792	4.946782	0.000000
76	6	0	-5.794113	5.000984	0.000000
77	1	0	-8.947591	3.808568	0.000000
78	1	0	-7.772112	5.844557	0.000000
79	6	0	-7.228035	-2.517357	0.000000
80	6	0	-5.835351	-2.571609	0.000000
81	6	0	-5.144754	-3.767758	0.000000
82	6	0	-5.794113	-5.000984	0.000000
83	1	0	-5.250103	-1.673774	0.000000
84	1	0	-4.074582	-3.709836	0.000000
85	6	0	-12.326019	1.188353	0.000000
86	6	0	-13.709776	1.190458	0.000000
87	1	0	-11.830806	2.138623	0.000000
88	1	0	-14.234494	2.129258	0.000000
89	6	0	-7.877435	-3.750554	0.000000
90	6	0	-7.186792	-4.946782	0.000000
91	1	0	-8.947591	-3.808568	0.000000

92	1	0	-7.772112	-5.844557	0.000000
93	6	0	-5.835351	2.571609	0.000000
94	6	0	-5.144754	3.767758	0.000000
95	1	0	-5.250103	1.673774	0.000000
96	1	0	-4.074582	3.709836	0.000000
97	6	0	-7.885855	-11.277786	0.000000
98	6	0	-7.192153	-10.080470	0.000000
99	6	0	-5.796430	-10.039711	0.000000
100	1	0	-8.961239	-11.262804	0.000000
101	1	0	-7.767505	-9.176467	0.000000
102	6	0	-5.823921	-12.468244	0.000000
103	6	0	-5.133866	-11.268822	0.000000
104	1	0	-5.273255	-13.392062	0.000000
105	1	0	-4.063301	-11.315090	0.000000
106	6	0	13.709776	-1.190458	0.000000
107	6	0	12.326019	-1.188353	0.000000
108	6	0	11.592859	0.000000	0.000000
109	1	0	14.234494	-2.129258	0.000000
110	1	0	11.830806	-2.138623	0.000000
111	6	0	13.709776	1.190458	0.000000
112	6	0	12.326019	1.188353	0.000000
113	1	0	14.234494	2.129258	0.000000
114	1	0	11.830806	2.138623	0.000000
115	6	0	5.044530	-8.737382	0.000000
116	6	0	5.043442	-6.300351	0.000000
117	6	0	2.934543	-7.517925	0.000000
118	6	0	-5.044530	-8.737382	0.000000
119	6	0	-2.934543	-7.517925	0.000000
120	6	0	-5.043442	-6.300351	0.000000
121	6	0	7.977985	-1.217574	0.000000
122	6	0	10.089060	0.000000	0.000000
123	6	0	7.977985	1.217574	0.000000
124	6	0	5.043442	6.300351	0.000000
125	6	0	5.044530	8.737382	0.000000
126	6	0	2.934543	7.517925	0.000000
127	6	0	-2.934543	7.517925	0.000000
128	6	0	-5.044530	8.737382	0.000000
129	6	0	-5.043442	6.300351	0.000000
130	6	0	-7.977985	1.217574	0.000000
131	6	0	-7.977985	-1.217574	0.000000
132	6	0	-10.089060	0.000000	0.000000
133	6	0	14.414475	0.000000	0.000000
134	1	0	15.489566	0.000000	0.000000
135	6	0	7.207237	12.483301	0.000000
136	1	0	7.744783	13.414358	0.000000
137	6	0	-7.207237	12.483301	0.000000
138	1	0	-7.744783	13.414358	0.000000
139	6	0	-14.414475	0.000000	0.000000
140	1	0	-15.489566	0.000000	0.000000
141	6	0	-7.207237	-12.483301	0.000000

142	1	0	-7.744783	-13.414358	0.000000
143	6	0	7.207237	-12.483301	0.000000
144	1	0	7.744783	-13.414358	0.000000
145	6	0	9.368862	-1.190165	0.000000
146	1	0	9.900760	-2.114595	0.000000
147	6	0	9.368862	1.190165	0.000000
148	1	0	9.900760	2.114595	0.000000
149	6	0	7.305711	0.000000	0.000000
150	1	0	6.238954	0.000000	0.000000
151	6	0	5.715144	7.518589	0.000000
152	1	0	6.781673	7.517012	0.000000
153	6	0	3.653717	8.708755	0.000000
154	1	0	3.119087	9.631607	0.000000
155	6	0	3.652856	6.326931	0.000000
156	1	0	3.119477	5.403092	0.000000
157	6	0	-3.653717	8.708755	0.000000
158	1	0	-3.119087	9.631607	0.000000
159	6	0	-3.652856	6.326931	0.000000
160	1	0	-3.119477	5.403092	0.000000
161	6	0	-5.715144	7.518589	0.000000
162	1	0	-6.781673	7.517012	0.000000
163	6	0	-7.305711	0.000000	0.000000
164	1	0	-6.238954	0.000000	0.000000
165	6	0	-9.368862	1.190165	0.000000
166	1	0	-9.900760	2.114595	0.000000
167	6	0	-9.368862	-1.190165	0.000000
168	1	0	-9.900760	-2.114595	0.000000
169	6	0	-3.652856	-6.326931	0.000000
170	1	0	-3.119477	-5.403092	0.000000
171	6	0	-5.715144	-7.518589	0.000000
172	1	0	-6.781673	-7.517012	0.000000
173	6	0	-3.653717	-8.708755	0.000000
174	1	0	-3.119087	-9.631607	0.000000
175	6	0	3.653717	-8.708755	0.000000
176	1	0	3.119087	-9.631607	0.000000
177	6	0	5.715144	-7.518589	0.000000
178	1	0	6.781673	-7.517012	0.000000
179	6	0	3.652856	-6.326931	0.000000
180	1	0	3.119477	-5.403092	0.000000

Benzene

$E_0 = E_{elec} + ZPE$ = -230.595463

$H = E + RT$ = -230.590423

Center Number	Atomic Number	Atomic Type	Coordinates (Angstroms) X	Y	Z
1	6	0	0.000000	1.386175	0.000000
2	6	0	1.200463	0.693087	0.000000
3	1	0	0.000000	2.461761	0.000000
4	1	0	2.131948	1.230881	0.000000
5	6	0	-1.200463	-0.693087	0.000000
6	6	0	0.000000	-1.386175	0.000000
7	1	0	-2.131948	-1.230881	0.000000
8	1	0	0.000000	-2.461761	0.000000
9	6	0	-1.200463	0.693087	0.000000
10	1	0	-2.131948	1.230881	0.000000
11	6	0	1.200463	-0.693087	0.000000
12	1	0	2.131948	-1.230881	0.000000

1,3,5,2,4,6-trioxatriborinane

$E_0 = E_{elec} + ZPE$ = -300.642437

$H = E + RT$ = -300.636644

Center Number	Atomic Number	Atomic Type	Coordinates (Angstroms) X	Y	Z
1	8	0	0.000000	1.357069	0.000000
2	8	0	-1.175256	-0.678534	0.000000
3	8	0	1.175256	-0.678534	0.000000
4	5	0	0.000000	-1.370901	0.000000
5	1	0	0.000000	-2.552688	0.000000
6	5	0	-1.187235	0.685450	0.000000
7	1	0	-2.210693	1.276344	0.000000
8	5	0	1.187235	0.685450	0.000000
9	1	0	2.210693	1.276344	0.000000

Phenylboronic acid

$E_0 = E_{elec} + ZPE$ = -405.68057

$H = E + RT$ = -405.672141

Center Number	Atomic Number	Atomic Type	Coordinates (Angstroms) X	Y	Z
1	5	0	0.000000	0.000000	-1.730972
2	6	0	0.000000	0.000000	-0.163062
3	6	0	0.000000	1.196332	0.554668
4	6	0	0.000000	1.200556	1.939621
5	1	0	0.000000	2.129732	0.021291
6	1	0	0.000000	2.132702	2.476568
7	6	0	0.000000	-1.196332	0.554668
8	6	0	0.000000	-1.200556	1.939621
9	1	0	0.000000	-2.129732	0.021291
10	1	0	0.000000	-2.132702	2.476568
11	8	0	0.000000	-1.196581	-2.383831
12	1	0	0.000000	-1.159844	-3.328650
13	8	0	0.000000	1.196581	-2.383831
14	1	0	0.000000	1.159844	-3.328650
15	6	0	0.000000	0.000000	2.632642
16	1	0	0.000000	0.000000	3.708786

1,3,5-triazine

$E_0 = E_{elec} + ZPE$ = -278.624669

$H = E + RT$ = -278.61989

Center Number	Atomic Number	Atomic Type	Coordinates (Angstroms) X	Y	Z
1	7	0	1.172416	0.676895	0.000000
2	7	0	-1.172416	0.676895	0.000000
3	7	0	0.000000	-1.353790	0.000000
4	6	0	0.000000	1.279206	0.000000
5	1	0	0.000000	2.354192	0.000000
6	6	0	-1.107825	-0.639603	0.000000
7	1	0	-2.038790	-1.177096	0.000000
8	6	0	1.107825	-0.639603	0.000000
9	1	0	2.038790	-1.177096	0.000000

Benzonitrile

$E_0 = E_{elec} + ZPE$ = -322.332137

$H = E + RT$ = -322.325493

Center Number	Atomic Number	Atomic Type	Coordinates (Angstroms) X	Y	Z
1	6	0	0.000000	0.000000	0.599164
2	6	0	0.000000	1.207009	-0.091172
3	6	0	0.000000	1.202594	-1.474285
4	1	0	0.000000	2.133027	0.453002
5	1	0	0.000000	2.133349	-2.011221
6	6	0	0.000000	-1.207009	-0.091172
7	6	0	0.000000	-1.202594	-1.474285
8	1	0	0.000000	-2.133027	0.453002
9	1	0	0.000000	-2.133349	-2.011221
10	6	0	0.000000	0.000000	2.044350
11	7	0	0.000000	0.000000	3.180782
12	6	0	0.000000	0.000000	-2.164225
13	1	0	0.000000	0.000000	-3.239288

Hydrogen (H_2)

$E_0 = E_{elec} + ZPE$ = -1.116243

$H = E + RT$ = -1.112938

Center Number	Atomic Number	Atomic Type	Coordinates (Angstroms) X	Y	Z
1	1	0	0.000000	0.000000	0.364980
2	1	0	0.000000	0.000000	-0.364980

SI.1.2. Harmonic oscillator model of aromaticity (HOMA)

HOMA is structural based index for measuring aromaticity and it defined by Kruszewski and Krygowski [2] as following equation.

$$\mathrm{HOMA} = 1 - \frac{\alpha}{n}\sum_{i=1}^{n}\left(R_{opt} - R_i\right)^2$$

According to the equation, α, n, R_{opt} and R_i are empirical constant, the number of bonds determined, optimal value and individual bond length, respectively. The values of α and R_{opt} for C-C benzene ring are 257.7 and 1.388, respectively [3]. The value of n is 6 since phenyl ring (ring A) has 6 bonds. The values for R_i were obtained from our optimized structures which is listed in Table S1. HOMA = 1 indicates the system has all equal bond and full delocalization (benzene-like) whereas HOMA = 0 shows the system with completely localized system. Note that we were not able to measure the HOMA value of ring B since α and R_{opt} values for B-O bond are not available.

Table S1. The C-C bond length values for all 6 bond in ring A

Number	COF-1	CTF-1	TPG
1	1.38315	1.38100	1.38128
2	1.39519	1.39108	1.39373
3	1.39518	1.39117	1.39374
4	1.38310	1.38102	1.38119
5	1.39518	1.39117	1.39374
6	1.39519	1.39108	1.39373

SI.1.3. NICS-X-scan

The optimized structures were considered with the aim of finding aromatic property of the pore. First, COF-1, CTF-1 and TPG sheets were set perpendicular on z-axis, then ghost atoms (Bq) were situated from centre (0.0 Å) to near the pore wall (5.0 Å) with 0.1 Å distancing next each other along the x-axis [4]. Afterward, a NMR=GIAO calculation was implemented as same as optimization level and basis set, HF/6-31G(d). Consequently, isotropic values for Bq atoms (multiplied by -1) were recorded and were arranged in Table S2.

Table S2. $NICS_{iso}$ values for COF-1, CTF-1 and TPG over the x-axis

Distance from centre of pore (Å)	COF-1	CTF-1	TPG
5.0	2.0600	2.8316	2.4801
4.9	2.0038	2.7777	2.4549
4.8	1.9491	2.7077	2.4058
4.7	1.8959	2.6302	2.3432
4.6	1.8436	2.5502	2.2736
4.5	1.7921	2.4703	2.2005
4.4	1.7411	2.3918	2.1264
4.3	1.6906	2.3150	2.0528
4.2	1.6405	2.2398	1.9806
4.1	1.5910	2.1662	1.9106
4.0	1.5424	2.0942	1.8431
3.9	1.4949	2.0240	1.7785
3.8	1.4488	1.9557	1.7171
3.7	1.4043	1.8895	1.6588
3.6	1.3616	1.8258	1.6039
3.5	1.3208	1.7648	1.5523
3.4	1.2821	1.7067	1.5039
3.3	1.2454	1.6516	1.4586
3.2	1.2109	1.5997	1.4164
3.1	1.1785	1.5510	1.3770
3.0	1.1480	1.5054	1.3404
2.9	1.1196	1.4629	1.3064
2.8	1.0929	1.4233	1.2748
2.7	1.0681	1.3865	1.2454
2.6	1.0449	1.3523	1.2181
2.5	1.0233	1.3206	1.1927
2.4	1.0031	1.2912	1.1692
2.3	0.9843	1.2640	1.1473
2.2	0.9669	1.2388	1.1271
2.1	0.9506	1.2155	1.1082
2.0	0.9355	1.1939	1.0908

1.9	0.9215	1.1739	1.0746
1.8	0.9085	1.1555	1.0596
1.7	0.8965	1.1385	1.0458
1.6	0.8854	1.1229	1.0330
1.5	0.8752	1.1085	1.0212
1.4	0.8658	1.0953	1.0104
1.3	0.8571	1.0832	1.0005
1.2	0.8493	1.0723	0.9915
1.1	0.8422	1.0623	0.9833
1.0	0.8358	1.0534	0.9759
0.9	0.8300	1.0454	0.9694
0.8	0.8249	1.0383	0.9635
0.7	0.8205	1.0322	0.9584
0.6	0.8167	1.0269	0.9540
0.5	0.8134	1.0224	0.9503
0.4	0.8108	1.0188	0.9473
0.3	0.8088	1.0160	0.9450
0.2	0.8074	1.0140	0.9433
0.1	0.8065	1.0128	0.9423
0.0	0.8062	1.0124	0.9420

SI.1.4. Partial atomic charges

The cluster structures used for calculation of partial atomic charges are presented in Figure S1, S2 and S3. The main parts of the solid sheets including one pore, the 6 trihedral connections resulting rings B as well as rings A were completely covered. Energy calculation using POP=Chelpg keyword was carried out at level of B3LYP with basis set of 6-31+G(d) [5]. The values for partial atomic charges obtained from electrostatic potential (EPS) fit were assigned for molecular dynamics (MD) simulations (Table S3, S4 and S5).

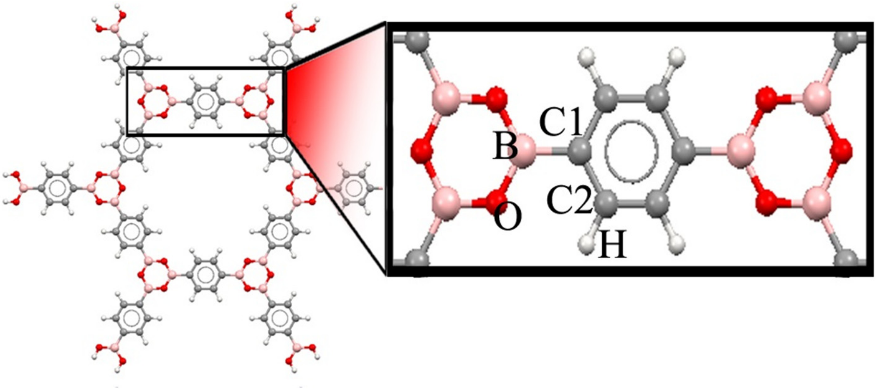

Figure S1. COF-1 cluster structure applied for calculation of partial atomic charges. Oxygen, boron, carbon and hydrogen atoms are displayed in red, pink, gray and white spheres, respectively.

Table S3. Partial atomic charges values for COF-1 as given by ESP-fit based on B3LYP/6-31+G(d) method

**Atom**	O	B	C1	C2	H
**Charge (e)**	-0.7474	0.7793	-0.1960	-0.0920	0.1038

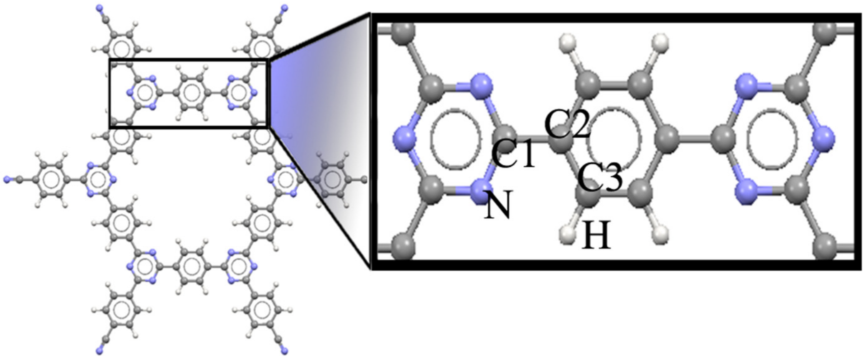

Figure S2. CTF-1 cluster structure applied for calculation of partial atomic charges. Nitrogen, carbon and hydrogen atoms are displayed in blue, gray and white spheres, respectively.

Table S4. Partial atomic charges values for CTF-1 as given by ESP-fit based on B3LYP/6-31+G(d) method

**Atom**	N	C1	C2	C3	H
**Charge (e)**	-0.6869	0.6486	-0.0559	-0.0537	0.1045

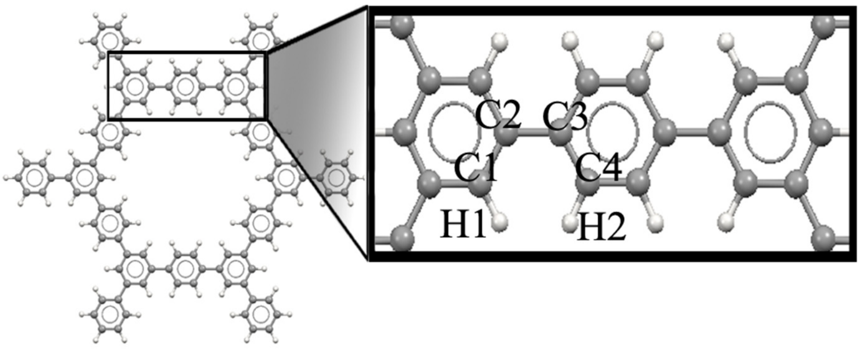

Figure S3. TPG cluster structure applied for calculation of partial atomic charges. Carbon and hydrogen atoms are displayed in gray and white spheres, respectively

Table S5. Partial atomic charges values for TPG as given by ESP-fit based on B3LYP/6-31+G(d) method

**Atom**	C1	C2	C3	C4	H1
**Charge (e)**	-0.1226	0.0298	0.1658	-0.1812	0.0493
**Atom**	H2				
**Charge (e)**	0.1293				

SI.2.1. Super cell and unit cell of the solid sheets

2×2×1 super cell structures and the relevant unit cell for COF-1, CTF-1 and TPG are shown in Figure S4, S5, S6, respectively. However, 1×1×1 extended super cell structures have been used for this study.

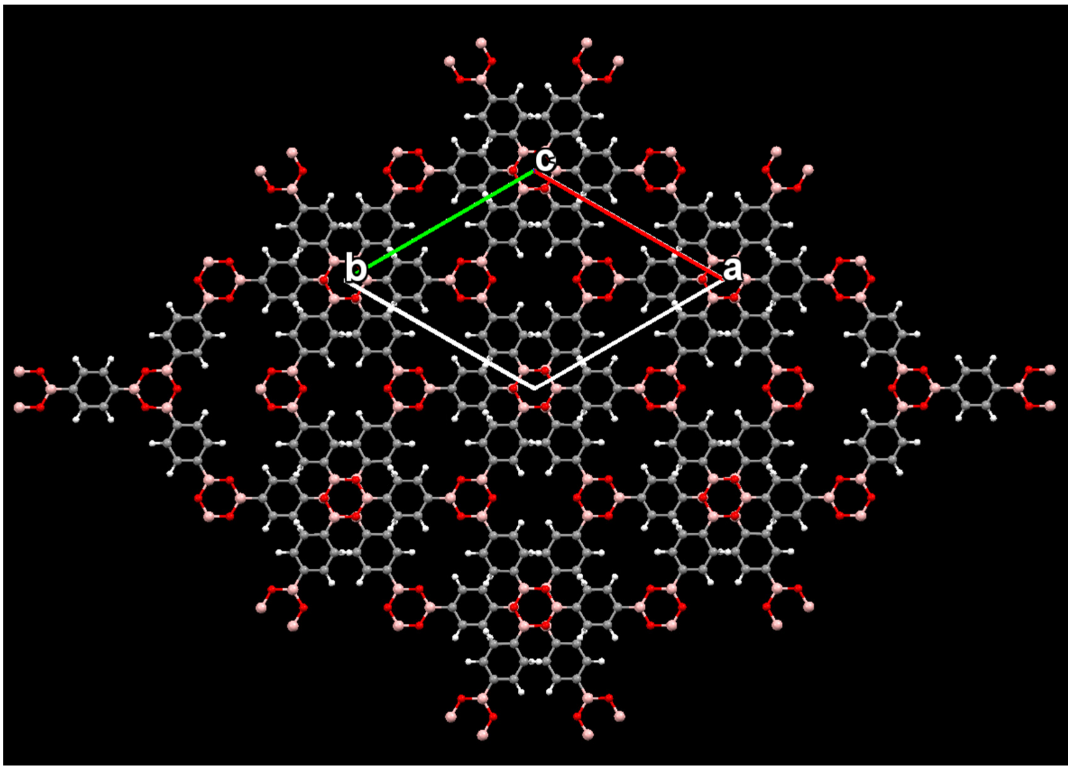

Figure S4. 2×2×1 extended super cell for COF-1 sheet

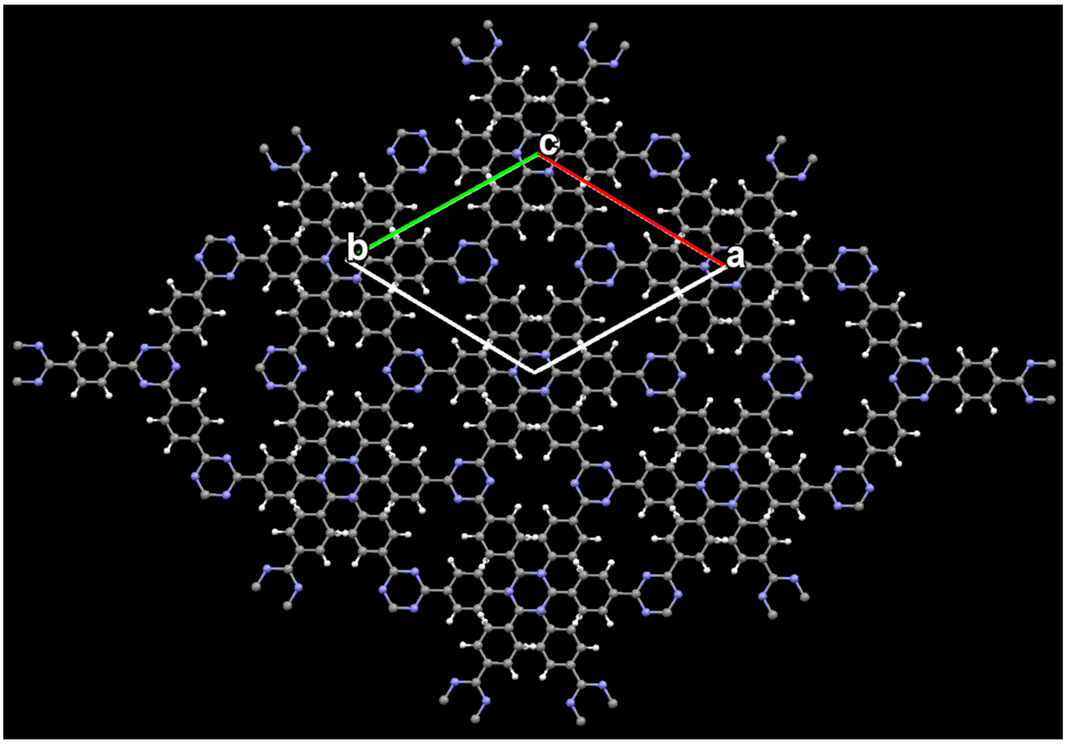

Figure S5. 2×2×1 extended super cell for CTF-1 sheet

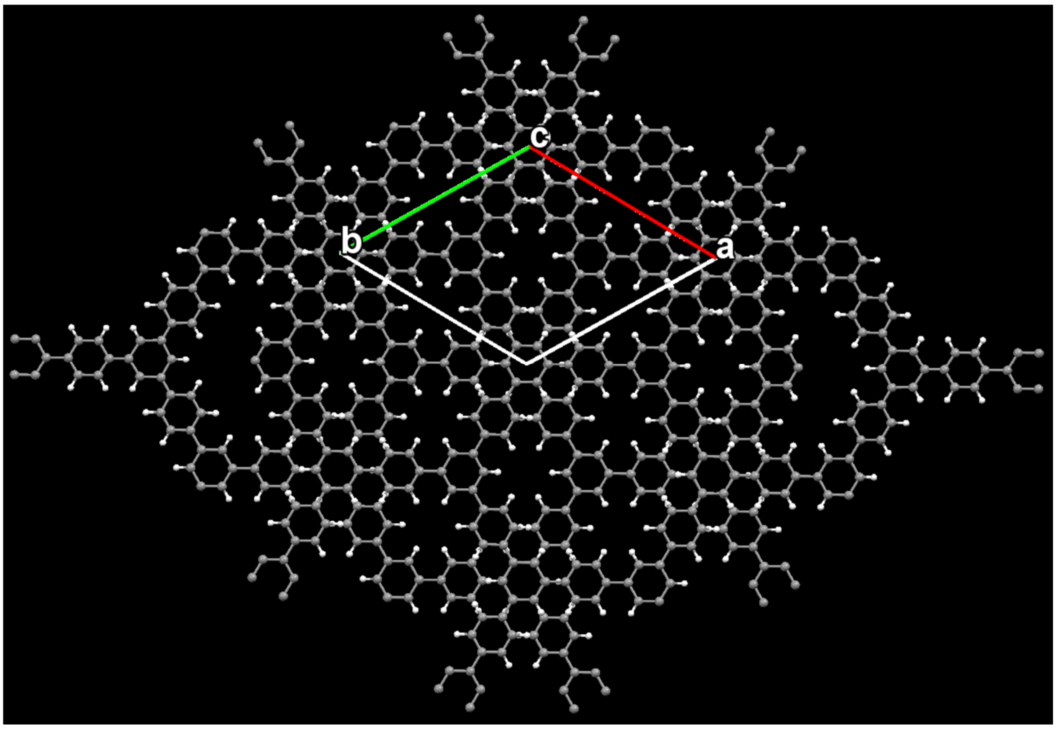

Figure S6. 2×2×1 extended super cell for TPG sheet

SI.2.2. Potential parameters

Table S6. Lennard-Jones potential parameters for COF-1, CTF-1 and TPG taken from OBGMX web-based server derived by UFF force field [6]

COF	Atom type	σ (nm)	ε (kj/mol)
COF-1	B_2	0.3638	0.7536
	O_2	0.3118	0.2512
	C_R	0.3431	0.4396
	H_	0.2571	0.1842
	O_3	0.3118	0.2512
CTF-1	C_R	0.3431	0.4396
	C_2	0.3431	0.4396
	N_R	0.3261	0.2889
	N_2	0.3261	0.2889
	H_	0.2571	0.1842
	C_3	0.3431	0.4396
TPG	C_R	0.3431	0.4396
	C_2	0.3431	0.4396
	H_	0.2571	0.1842
	C_3	0.3431	0.4396

SI.2.3. Total energy

Maximum number of steps for energy minimization of the systems was determined by 50000 steps. The algorithm of steepest descent minimization was applied, and the run was stopped at 30674, 50000 and 50000 steps with the potential energy of -6.43 x 10^4, -6.60 x 10^4 and -6.48 x 10^4 kJ/mol for COF-1, CTF-1 and TPG, respectively. MD production of the systems was continued for 50 ns, after they were equilibrated at 300 K using NVT ensemble and at pressure of 1 bar using NPT ensemble. As plotted in Figure S7, the total energy values of the systems are not severely fluctuated, thus dynamics behavior of the water and ethanol molecules on the solid sheets can be calculated.

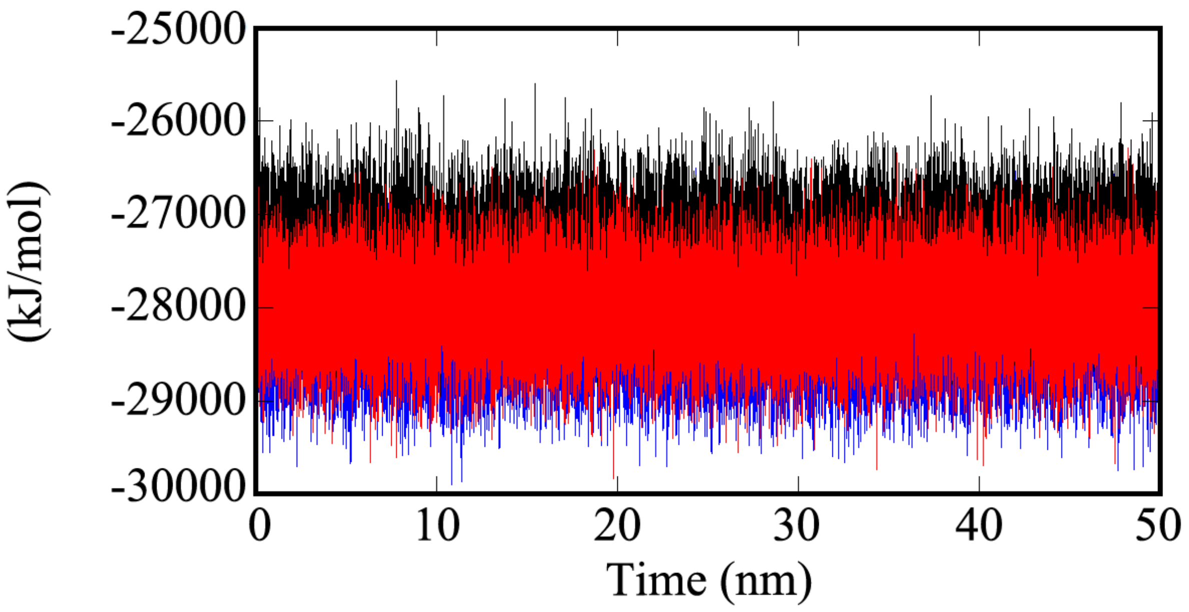

Figure S7. Calculated total energy of the systems; red, blue and black colors are represented for COF-1, CTF-1 and TPG

SI.2.4. Radial distribution function (RDF)

RDF is important tool for exploring the interaction of the considered molecules with various atoms on the surface of the solid sheets [7]. The RDF, g_{ij} (r), is defined as the following equation:

$$g_{ij}(r) = \frac{N_{ij}\ (r, r + \Delta r).V}{4\pi r^2.\Delta r.N_i.N_j}$$

According to the equation N_{ij}(r, r+Δr) is the number of atom j around atom i within the area by the distance between r and r+Δr. Also, V is indicated for the volume of system and N is the number of atoms. All the calculated RDF graphs for COF-1, CTF-1 and TPG are shown in Figure S8, S9 and S10. Also, the maximum peak value and its distance (nm) for each RDF graph can be found in Table S7.

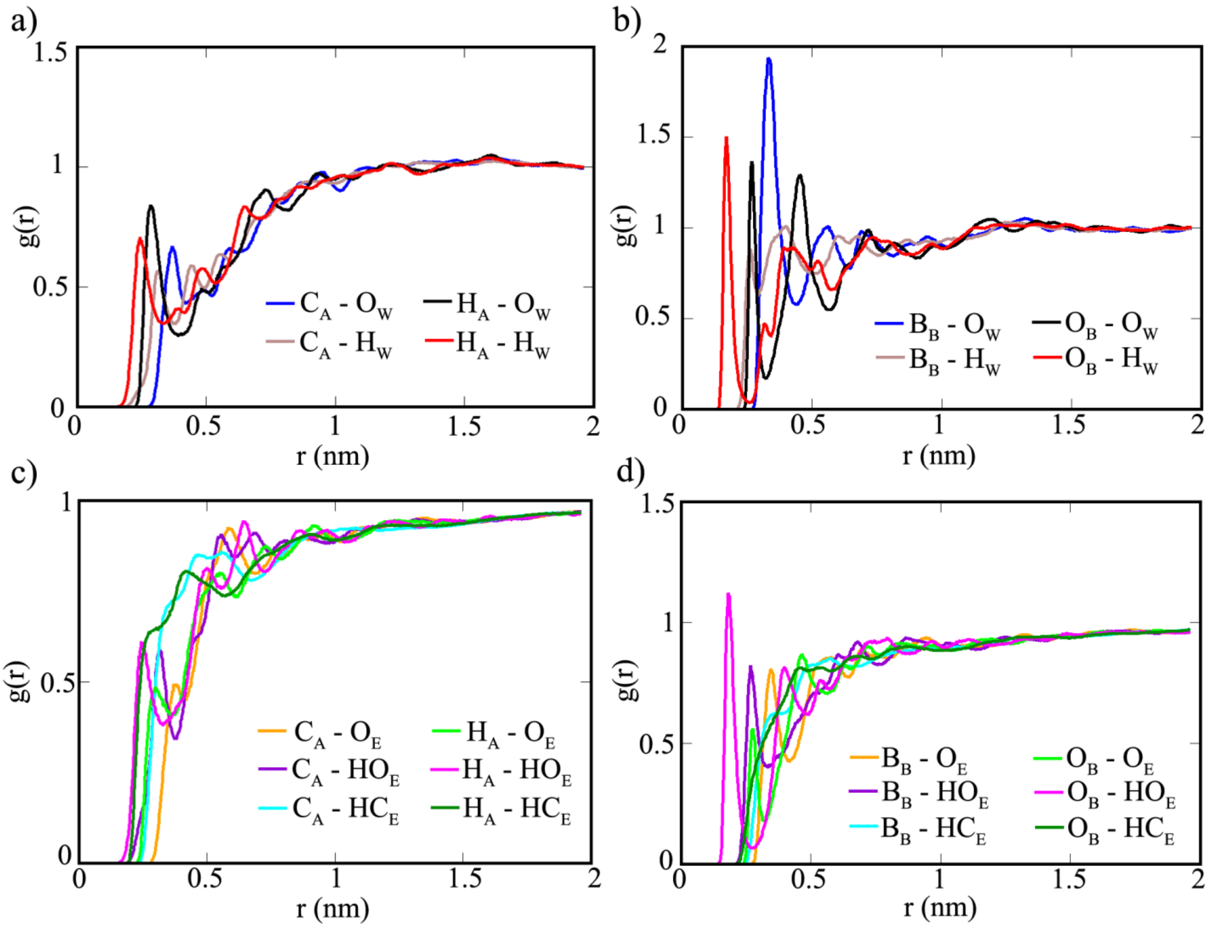

Figure S8. RDF graphs for COF-1; a) between atoms in ring A and atoms in water, b) between atoms in ring B and atoms in water, c) between atoms in ring A and atoms in ethanol, and d) between atoms in ring B and atoms in ethanol

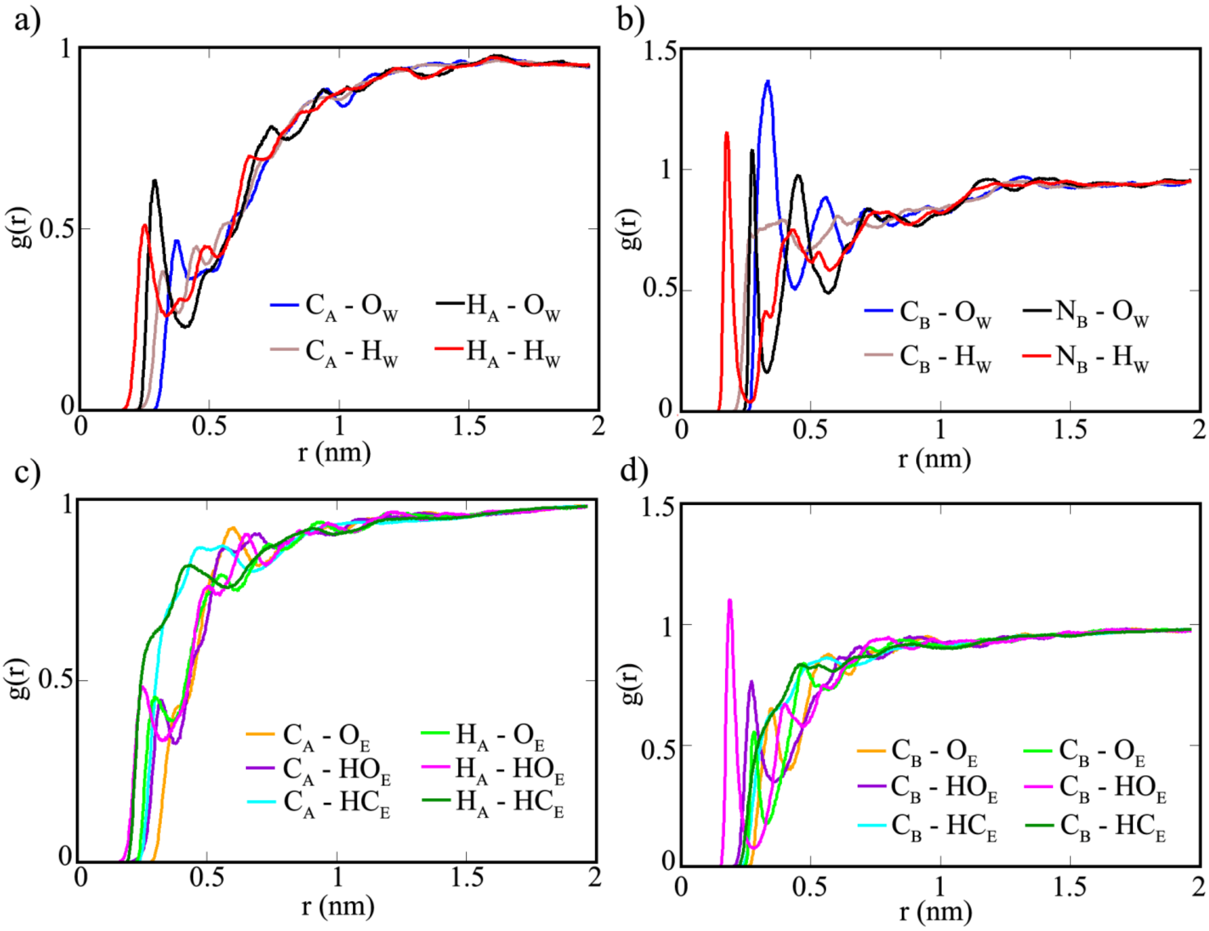

Figure S9. RDF graphs for CTF-1; a) between atoms in ring A and atoms in water, b) between atoms in ring B and atoms in water, c) between atoms in ring A and atoms in ethanol, and d) between atoms in ring B and atoms in ethanol

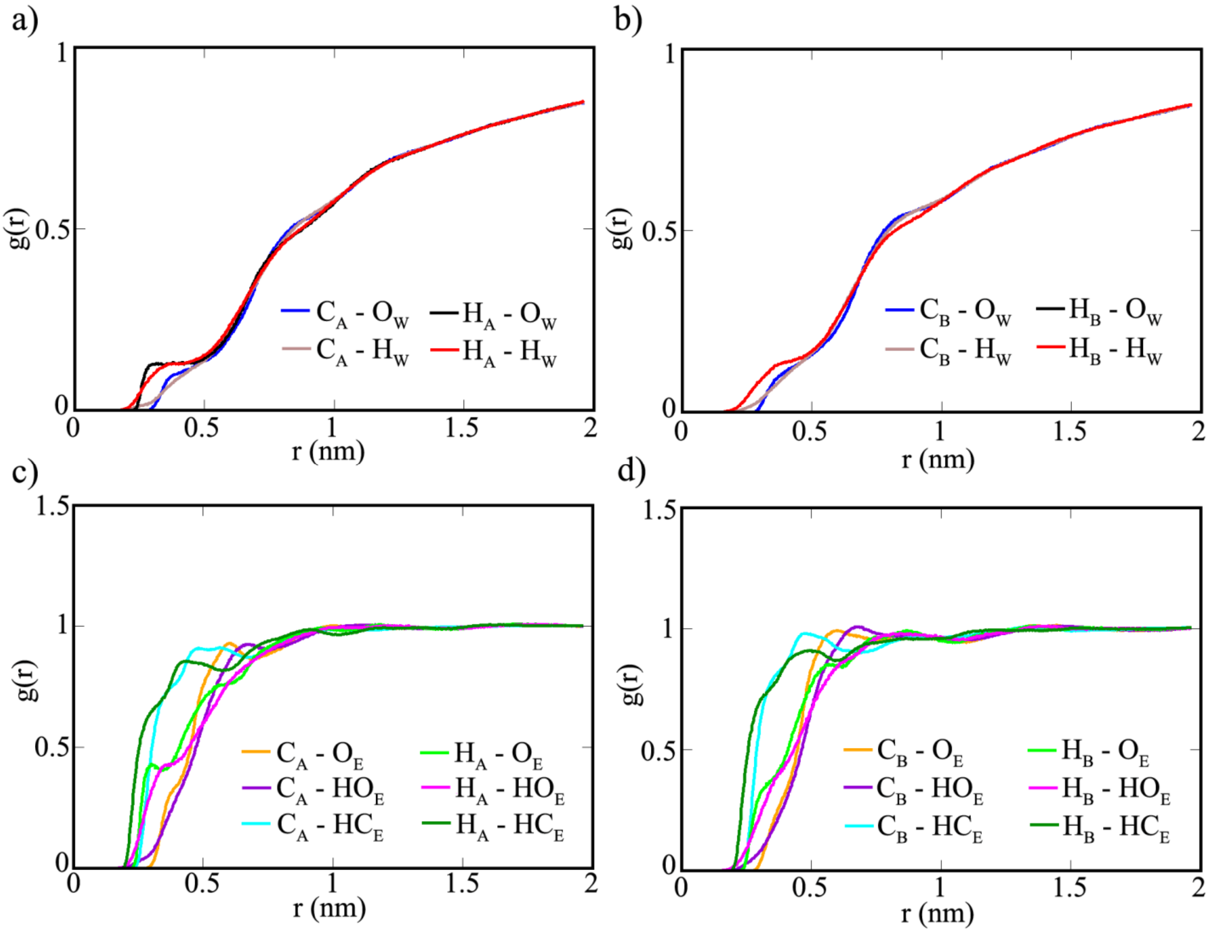

Figure S10. RDF graphs for TPG; a) between atoms in ring A and atoms in water, b) between atoms in ring B and atoms in water, c) between atoms in ring A and atoms in ethanol, and d) between atoms in ring B and atoms in ethanol

Table S7. The maximum peak and its corresponding distance for each RDF

COF-1	CTF-1	TPG
**Ring A – water**	**Ring A – water**	**Ring A – water**
C_A – O_{water} [1.03 in 1.6 nm]	C_A – O_{water} [0.97 in 1.6 nm]	C_A – O_{water} [0.85 in 1.9 nm]
C_A – H_{water} [1.02 in 1.6 nm]	C_A – H_{water} [0.96 in 1.6 nm]	C_A – H_{water} [0.85 in 1.9 nm]
H_A – O_{water} [1.05 in 1.6 nm]	H_A – O_{water} [0.98 in 1.6 nm]	H_A – O_{water} [0.85 in 1.9 nm]
H_A – H_{water} [1.04 in 1.6 nm]	H_A – H_{water} [0.97 in 1.6 nm]	H_A – H_{water} [0.85 in 1.9 nm]
**Ring B – water**	**Ring B – water**	**Ring B – water**
B_B – O_{water} [1.94 in 0.3 nm]	C_B – O_{water} [1.37 in 0.3 nm]	C_B – O_{water} [0.84 in 1.9 nm]
B_B – H_{water} [1.03 in 1.2 nm]	C_B – H_{water} [0.95 in 1.3 nm]	C_B – H_{water} [0.84 in 1.9 nm]
O_B – O_{water} [1.37 in 0.3 nm]	N_B – O_{water} [1.08 in 0.3 nm]	H_B – O_{water} [0.84 in 1.9 nm]
O_B – H_{water} [1.50 in 0.2 nm]	N_B – H_{water} [1.15 in 0.2 nm]	H_B – H_{water} [0.84 in 1.9 nm]
**Ring A – ethanol**	**Ring A – ethanol**	**Ring A – ethanol**
C_A – $O_{ethanol}$ [0.97 in 1.9 nm]	C_A – $O_{ethanol}$ [0.98 in 1.9 nm]	C_A – $O_{ethanol}$ [1.00 in 1.7 nm]
C_A – $HO_{ethanol}$ [0.97 in 1.9 nm]	C_A – $HO_{ethanol}$ [0.98 in 1.9 nm]	C_A – $HO_{ethanol}$ [1.00 in 1.7 nm]
C_A – $HC_{ethanol}$ [0.97 in 1.9 nm]	C_A – $HC_{ethanol}$ [0.98 in 1.9 nm]	C_A – $HC_{ethanol}$ [1.00 in 1.7-1.9 nm]
H_A – $O_{ethanol}$ [0.97 in 1.9 nm]	H_A – $O_{ethanol}$ [0.98 in 1.9 nm]	H_A – $O_{ethanol}$ [1.00 in 1.7 nm]
H_A – $HO_{ethanol}$ [0.97 in 1.8 nm]	H_A – $HO_{ethanol}$ [0.98 in 1.9 nm]	H_A – $HO_{ethanol}$ [1.00 in 1.7-1.8 nm]
H_A – $HC_{ethanol}$ [0.97 in 1.9 nm]	H_A – $HC_{ethanol}$ [0.98 in 1.9 nm]	H_A – $HC_{ethanol}$ [1.00 in 1.6 nm]
**Ring B – ethanol**	**Ring B – ethanol**	**Ring B – ethanol**
B_B – $O_{ethanol}$ [0.97 in 1.7 nm]	C_B – $O_{ethanol}$ [0.98 in 1.7 nm]	C_B – $O_{ethanol}$ [1.01 in 1.4 nm]
B_B – $HO_{ethanol}$ [0.97 in 1.7 nm]	C_B – $HO_{ethanol}$ [0.98 in 1.7 nm]	C_B – $HO_{ethanol}$ [1.01 in 1.4 nm]
B_B – $HC_{ethanol}$ [0.97 in 1.9 nm]	C_B – $HC_{ethanol}$ [0.98 in 1.7 nm]	C_B – $HC_{ethanol}$ [1.01 in 1.3 nm]
O_B – $O_{ethanol}$ [0.97 in 1.8 nm]	N_B – $O_{ethanol}$ [0.98 in 1.8 nm]	H_B – $O_{ethanol}$ [1.01 in 1.3 nm]
O_B – $HO_{ethanol}$ [1.12 in 0.2 nm]	N_B – $HO_{ethanol}$ [1.10 in 0.2 nm]	H_B – $HO_{ethanol}$ [1.01 in 1.3 nm]
O_B – $HC_{ethanol}$ [0.97 in 1.9 nm]	N_B – $HC_{ethanol}$ [0.98 in 1.9 nm]	H_B – $HC_{ethanol}$ [1.01 in 1.9 nm]

SI.2.5. Average number of hydrogen bond (HB) interaction

Table S8.

**Surfaces**	**Ring/solvent**	**Standard deviation**	**HB interaction**
COF-1	Ring A/Ethanol	0.00	0.00±0.00
	Ring B/Ethanol	1.75×10^{-2}	8.70±0.28
	Ring A/Water	0.00	0.00±0.00
	Ring B/Water	2.17×10^{-2}	17.03±0.49
CTF-1	Ring A/Ethanol	0.00	0.00±0.00
	Ring B/Ethanol	1.83×10^{-2}	9.00±0.25
	Ring A/Water	0.00	0.00±0.00
	Ring B/Water	2.46×10^{-2}	14.82±0.71
TPG	Ring A/Ethanol	0.00	0.00±0.00
	Ring B/Ethanol	0.00	0.00±0.00
	Ring A/Water	0.00	0.00±0.00
	Ring B/Water	0.00	0.00±0.00

SI.2.6. Partial density

Table S9. The maximum and minimum values of partial density as well as their corresponding distance from centre of z-axis in COF-1, CTF-1 and TPG

Solvent	COF-1	CTF-1	TPG
**Ethanol**	Max:<br>748 kg.m^{-3} (0.49 nm)<br>756 kg.m^{-3} (-0.49 nm)<br>Min:<br>250 kg.m^{-3} (0.07 nm)<br>243 kg.m^{-3} (-0.07 nm)	Max:<br>769 kg.m^{-3} (0.49 nm)<br>744 kg.m^{-3} (-0.49 nm)<br>Min:<br>259 kg.m^{-3} (0.07 nm)<br>259 kg.m^{-3} (-0.07 nm)	Max:<br>832 kg.m^{-3} (0.49 nm)<br>813 kg.m^{-3} (-0.49 nm)<br>Min:<br>263 kg.m^{-3} (0.07 nm)<br>261 kg.m^{-3} (-0.07 nm)
**Water**	Max:<br>265 kg.m^{-3} (0.91 nm)<br>267 kg.m^{-3} (-0.91 nm)<br>Min:<br>153 kg.m^{-3} (0.21 nm)<br>158 kg.m^{-3} (-0.21 nm)	Max:<br>269 kg.m^{-3} (0.91 nm)<br>265 kg.m^{-3} (2.89 nm)<br>Min:<br>127 kg.m^{-3} (0.21 nm)<br>134 kg.m^{-3} (-0.21 nm)	Max:<br>292 kg.m^{-3} (3.43 nm)<br>290 kg.m^{-3} (-3.43 nm)<br>Min:<br>35 kg.m^{-3} (0.07 nm)<br>37 kg.m^{-3} (-0.07 nm)

SI.2.7. Mean square displacement (MSD)

Table S10.

Surfaces	MSD of ethanol (10^{-5} cm^2 s^{-1})	Standard deviation	MSD of water (10^{-5} cm^2 s^{-1})	Standard deviation
COF-1	0.61±0.07	1.50×10^{-1}	0.80±0.01	1.95×10^{-1}
CTF-1	0.67±0.02	1.64×10^{-1}	0.82±0.02	1.99×10^{-1}
TPG	0.57±0.02	1.40×10^{-1}	0.69±0.02	1.67×10^{-1}

References

1. Baranac-Stojanovic, M. Cyclic π Electron Delocalization in Fluoroborazines. *J. Phys. Chem. A* **2013**, *117*, 11540–11547. [doi.org/10.1021/jp407454x]

2. Kruszewski, J.; Krygowski, T.M. Definition of aromaticity basing on the harmonic oscillator model. *Tetrahedron Lett.* **1972**, *13*, 3839–3842. [doi.org/10.1016/S0040-4039(01)94175-9]

3. Fallah-Bagher-Shaidaei, H.; Farkhonde, R.; Navideh, L.G. Aromaticity of topological isomers of cyclazines and their [14] to [18] annulenes. *Comput. Theor. Chem.* **2011**, *963*, 525–532. [doi.org/10.1016/j.comptc.2010.11.027]

4. Schaffroth, M.; Gershoni-Poranne, R.; Stanger, A.; Bunz, U.H.F. Tetraazaacenes Containing Four-Membered Rings in Different Oxidation States. Are They Aromatic? A Computational Study. *J. Org. Chem.* **2014**, *79*, 11644–11650. [doi.org/10.1021/jo502297w]

5. Kotzabasaki, M.; Galdadas, I.; Tylianakis, E.; Klontzas, E.; Cournia, Z.; Froudakis, G.E. Multiscale simulations reveal IRMOF-74-III as a potent drug carrier for gemcitabine delivery. *J. Mater. Chem. B* **2017**, *5*, 3277–3282. [doi.org/10.1039/C7TB00220C]

6. Garberoglio, G. OBGMX: A web-based generator of GROMACS topologies for molecular and periodic systems using the universal force field. *J. Comput. Chem.* **2012**, *33*, 2204–2208. [doi.org/10.1002/jcc.23049]

7. Xu, P.; Lang, X.; Fan, S.; Wang, Y.; Chen, J. Molecular dynamics simulation of methane hydrate growth in the presence of the natural product pectin. *J. Phys. Chem. C* **2016**, *120*, 5392–5397. [doi.org/10.1021/acs.jpcc.5b10342]
